# Ferroptosis-driven in situ vaccine-like antitumor effects: NIR-triggered IFBM hydrogel synergizes with sorafenib to unleash systemic antitumor immunity

**DOI:** 10.1186/s12951-026-04262-z

**Published:** 2026-03-08

**Authors:** Zilong Jiang, Rui Fu, Pengping Li, Jiawei Zhang, Yixian Cheng, Kang Yang, Junjie Chen, Yaqi Zhang, Lihua Su, Yu Lei, Bo Chen, Guodong Cao

**Affiliations:** 1 Department of General Surgery, The First Affiliated Hospital of Anhui Medical University, Hefei, 230022 Anhui Province China; 2 Department of Oncology, The First Affiliated Hospital of Anhui Medical University, Hefei, 230022 Anhui Province China; 3 The People’s Hospital of Xiaoshan District, Xiaoshan Affiliated Hospital of Wenzhou Medical University, Hangzhou, China; 4 Department of Gastroenterology, Lishui Municipal Central Hospital, The Fifth Affiliated Hospital of Wenzhou Medical University, Lishui, China; 5 The Endocrinology Department, The First Affiliated Hospital of Anhui Medical University, Hefei, 230022 Anhui Province China

**Keywords:** Ferroptosis, Immunotherapy, Photothermal Therapy, In Situ Vaccine, Combination Therapy, Sorafenib

## Abstract

The immunosuppressive tumor microenvironment and immune evasion mechanisms inhibit the development of effective antitumor responses and enduring immune memory, thereby diminishing the efficacy of cancer immunotherapy. In situ vaccines (ISVs) have been developed to overcome these challenges by inducing localized immunogenic cell death (ICD) and activating systemic immunity via dendritic cells (DCs) and CD8^+^ T cells. In this study, we engineered ferrous sulfide–bovine serum albumin–cell membrane nanoclusters to trigger ferroptosis, which were co-encapsulated with the photothermal agent indocyanine green in a thermosensitive hydrogel. This platform enables near-infrared-responsive drug release and works with sorafenib to boost ferroptosis. Mechanistic studies revealed that this combined regimen markedly enhanced DC-mediated antigen presentation and CD8^+^ T-cell infiltration, leading to substantial antitumor efficacy and complete eradication of distant liver metastases. Ultimately, the indocyanine green–ferrous sulfide–bovine serum albumin–cell membrane hydrogel–sorafenib system induces ferroptosis-driven ICD, promoting an ISV-like effect that activates systemic antitumor immunity and demonstrates strong therapeutic efficacy, suggesting its potential as a novel cancer treatment strategy.

**Supplementary Information:**

The online version contains supplementary material available at 10.1186/s12951-026-04262-z.

## Introduction

The immunosuppressive tumor microenvironment (TME) and immune evasion mechanisms substantially obstruct the generation of vigorous antitumor responses and lasting immune memory, consequently diminishing the overall efficacy of cancer immunotherapy [1]. Conventional treatment methods, such as surgery, radiotherapy, and chemotherapy, often fail to fully solve these challenges because they have non-specific effects and the potential for inducing resistance [2, 3]. In this context, In situ vaccines (ISVs) have emerged as a promising approach to overcome these barriers [4]. ISVs use the tumor as a source of endogenous antigen by causing localized immunogenic cell death (ICD), which releases damage-associated molecular patterns (DAMPs) and tumor-associated antigens (TAAs) [5, 6]. This process activates dendritic cells (DCs) and CD8^+^ T cells, which helps the body fight cancer and creates a long-term antigen reservoir to prevent recurrence and metastasis [7].

Ferroptosis, an iron-dependent regulated cell death marked by the accumulation of lipid peroxides and disruption of cell membrane integrity, is a vital entry point for activating anticancer immunity within the ISV framework [8, 9]. Ferroptosis directly eradicates tumor cells and also greatly increases ICD by enhancing the release of DAMPs [10]. This results in enhanced DC maturation, antigen cross-presentation, and CD8^+^ T-cell infiltration, effectively converting immunologically “cold” tumors into “hot” tumors that exhibit greater susceptibility to immune attack [11]. Recent studies highlight ferroptosis as a modulator that connects tumor cell death with immune activation, presenting synergistic potential with immunotherapies [12].

Sorafenib (Sor), a multi-kinase inhibitor approved for advanced hepatocellular carcinoma (HCC), illustrates the therapeutic potential of ferroptosis induction by downregulating glutathione peroxidase 4 (GPX4) and exacerbating lipid peroxidation [13]. However, Sor monotherapy is limited by low intratumoral iron level, poor bioavailability, and rapid clearance, which makes ferroptosis less effective and limits antitumor effects [14]. To overcome these limitations, we developed the indocyanine green–ferrous sulfide–bovine serum albumin–cell membrane (IFBM) hydrogel system, which includes ferrous sulfide@bovine serum albumin@cell membrane (FeS@BSA@CM) nanoclusters (NCs). These NCs provide catalytic iron to work with Sor, which increases lipid peroxidation and overcomes resistance mechanisms. This iron-supplemented, ferroptosis-driven strategy improved the antitumor effects in our study, consistent with previous reports [15].

Furthermore, IFBM platform uses indocyanine green (ICG) as a photothermal agent in a temperature-sensitive hydrogel matrix. This was proposed as an advanced drug delivery platform that allows near-infrared (NIR)-responsive controlled drug release [16, 17]. When injected into a tumor, the hydrogel creates a stable depot at body temperature (37 °C), and NIR irradiation activates ICG to generate localized hyperthermia (> 43 °C), which breaks down the hydrogel and releases FeS@BSA@CM (FBM) NCs as needed. This stimuli-responsive design facilitates precise spatiotemporal control of drug delivery, but it combines photothermal therapy (PTT) with ferroptosis, further releasing DAMPs and TAAs to enhance the immunogenic environment [18].

The IFBM hydrogel–Sor system causes ferroptosis-driven ICD, which has an ISV-like effect that boosts systemic antitumor immunity. Mechanistic studies revealed improved DC-mediated antigen presentation, increased CD8^+^ T-cell infiltration, and potent suppression of primary tumors, resulting in total eradication of distant liver metastases. This multifaceted strategy signifies a promising approach for cancer treatment, using ferroptosis-ICD synergy to inhibit metastatic progression and induce lasting immune memory.

## Results

### Preparation and characterization of different NCs and gels

**Fig. 1:**
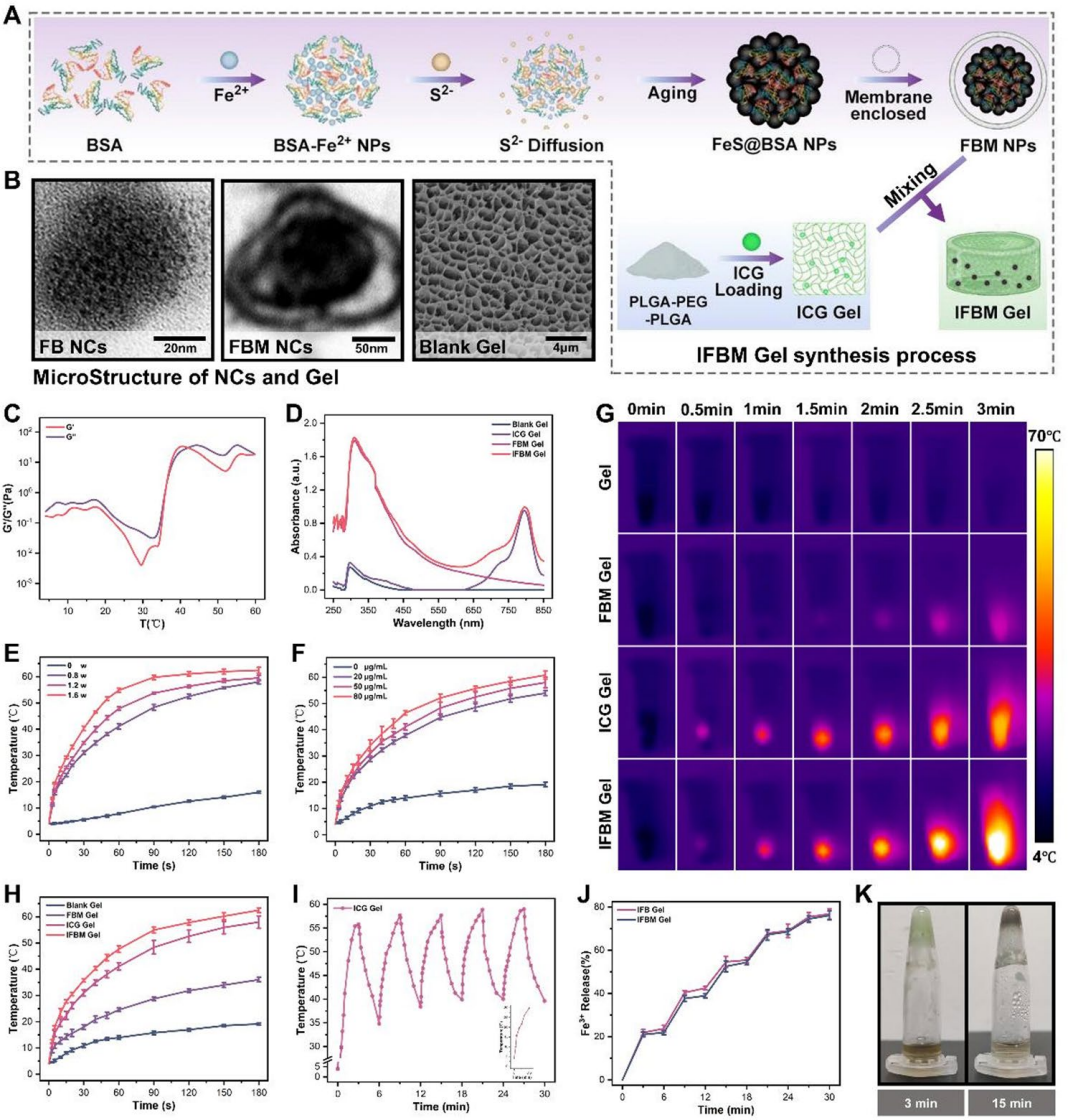
**A**) Schematic diagram of the preparation process of IFBM gel. **B**) Transmission electron micrographs of FB NCs, FBM NCs, and scanning electron micrographs of PPP gel (from left to right). **C**) Rheological properties of IFBM gel at 4–60 °C. **D**) Spectral absorption curves of different component hydrogels (with error bars in Fig. S3A). **E**) Temperature-time change curves of 50 µg/mL ICG gel under NIR irradiation at different powers. **F**) Temperature-time change curves of different concentrations of ICG gel under 1.2 W/cm^2^ NIR irradiation. **G**) Thermal imaging photographs of different components of hydrogel under 1.2 W/cm^2^ NIR irradiation, and **H**) is the corresponding temperature-time change curve. **I**) Temperature-time change curves of 50 µg/mL ICG gel under 1.2 W/cm^2^ NIR on-off irradiation (with error bars in Fig. S3B). **J**) Fe^3+^ release curve of IFBM gel under on-off irradiation. **K**) Degradation images of IFBM gel under 1.2 W/cm^2^ NIR irradiation: left side irradiated for 3 min, right side irradiated for 15 min. Data are presented as mean ± SD (*n* = 3). *p < 0.05, **p < 0.01, ***p < 0.001, and ****p < 0.0001

First, ferrous sulfide–bovine serum albumin (FB) NCs were synthesized using self‑assembly. Subsequently, cell‑membrane encapsulation was used to make FBM NCs, and IFBM gel was obtained by co‑loading FBM NCs and ICG into the PLGA–PEG–PLGA (PPP) thermosensitive gel (Fig. 1A). Transmission and scanning electron microscopy analyses were conducted to examine their microstructure (Fig. 1B). These analyses demonstrated that FB and FBM NCs were uniformly dispersed spherical particles, whereas the PPP gel exhibited a homogeneous, loose porous network, offering ample interstitial space for the integration of spherical NCs and consequently enhancing drug‑loading capacity. Several gel formulations were prepared (Fig. S1). To evaluate the thermoresponsive behavior, the samples were placed at different temperatures. At 4°C, the gel remained in a liquid state, which made it easier to inject and load drugs. When heated to 37°C, which is the normal body temperature, it quickly turned into a stable gel, which acted as a drug depot in the body. The gel melted when it was heated to 60°C, which caused the drug to be released on demand. Black FeS NCs progressively lose their characteristic color as they oxidize; hence, we monitored the oxidation process by recording the corresponding color changes (Figs. S2A-B). At 4°C, FBM gel exhibited slow fading over 4 h; however, it faded completely in approximately 30 min at 37°C, indicating that higher temperature accelerates the oxidation process significantly. These findings further demonstrated that NIR irradiation elevates the temperature of IFBM gel, consequently accelerating lipid oxidation and amplifying ferroptosis. Additionally, the time needed for complete oxidation in the gel (4 h at 4°C and 30 min at 37°C) was longer than that of the corresponding NC suspensions (3 h and 25 min, respectively; Figs. S2C-D). This suggests that the gel matrix slows down oxidation and allows for more controlled drug release. Rheological analysis (Fig. 1C) revealed that the storage modulus (G’) increased progressively with temperature and surpassed the loss modulus (G’’) near 37 °C, confirming that IFBM gel solidifies under physiological conditions and effectively entraps the NCs to create an in vivo drug depot. When the temperatures increased above 43 °C, the loss modulus (G’’) exceeded the storage modulus (G’), triggering on‑demand release of the encapsulated drug and highlighting the gel’s excellent controlled‑release performance. Subsequently, this study performed the ultraviolet–visible spectroscopy (Fig. 1D), which revealed distinct absorption maxima at ~ 790 nm for ICG gel and ~ 303 nm for FBM gel, whereas IFBM gel exhibited both peaks, confirming successful integration of the two components.

The photothermal performance of the gels was subsequently assessed by evaluating the heating behavior of the ICG gel under different NIR power densities and ICG concentrations (Figs. 1E-F). As the laser power or the ICG content increased, the maximum temperature increased accordingly, reaching approximately 60 °C at the highest settings. Subsequently, we tested the photothermal performance of the different gel formulations. Figure 1G-H depict the thermographs and temperature-rise curves. The blank gel exhibited negligible temperature change, whereas FBM gel exhibited modest photothermal conversion that was weaker than that of the ICG gel. Consequently, the IFBM gel, which has both photothermal components, reached a significantly higher temperature (> 40 °C within 1 min under 1.2 W/cm^2^ NIR irradiation). The photothermal stability of IFBM gel was subsequently assessed. Figure 1I depicts that the ICG gel maintained a stable temperature profile through five consecutive NIR on/off cycles. This indicates that a single intratumoral dose can handle repeated irradiations. This study further examined the influence of ambient temperature and initial gel temperature on photothermal behavior (Fig. S3C); its peak temperature under NIR exposure remained largely unchanged regardless of whether the starting temperature was 4–37 °C. The *in vitro* drug-release profile was subsequently assessed. First, we established the absorbance–concentration calibration curve for Fe^3+^(Fig. S4A), and obtained a linear fit at the 303 nm absorption peak (Fig. S4B). Using this standard, we quantified the level of Fe^3+^ concentration in the supernatant from IFB gel and IFBM gel after each photothermal on/off cycle, and the corresponding release profiles were plotted (Fig. 1J). Fe^3+^ release exhibited strong on‑demand characteristics, with approximately 80% cumulative release after five cycles; cell membrane coating slightly slowed the release kinetics but exhibited minimal impact on the final cumulative amount. Photothermal‑induced degradation of IFBM gel was evaluated (Fig. 1K); continuous NIR irradiation for 15 min caused a marked reduction in gel volume compared with 3 min irradiation, indicating that the gel was easily photothermally degradable.

### In vitro cytotoxicity of IFBM gel combined with sorafinib

**Fig. 2:**
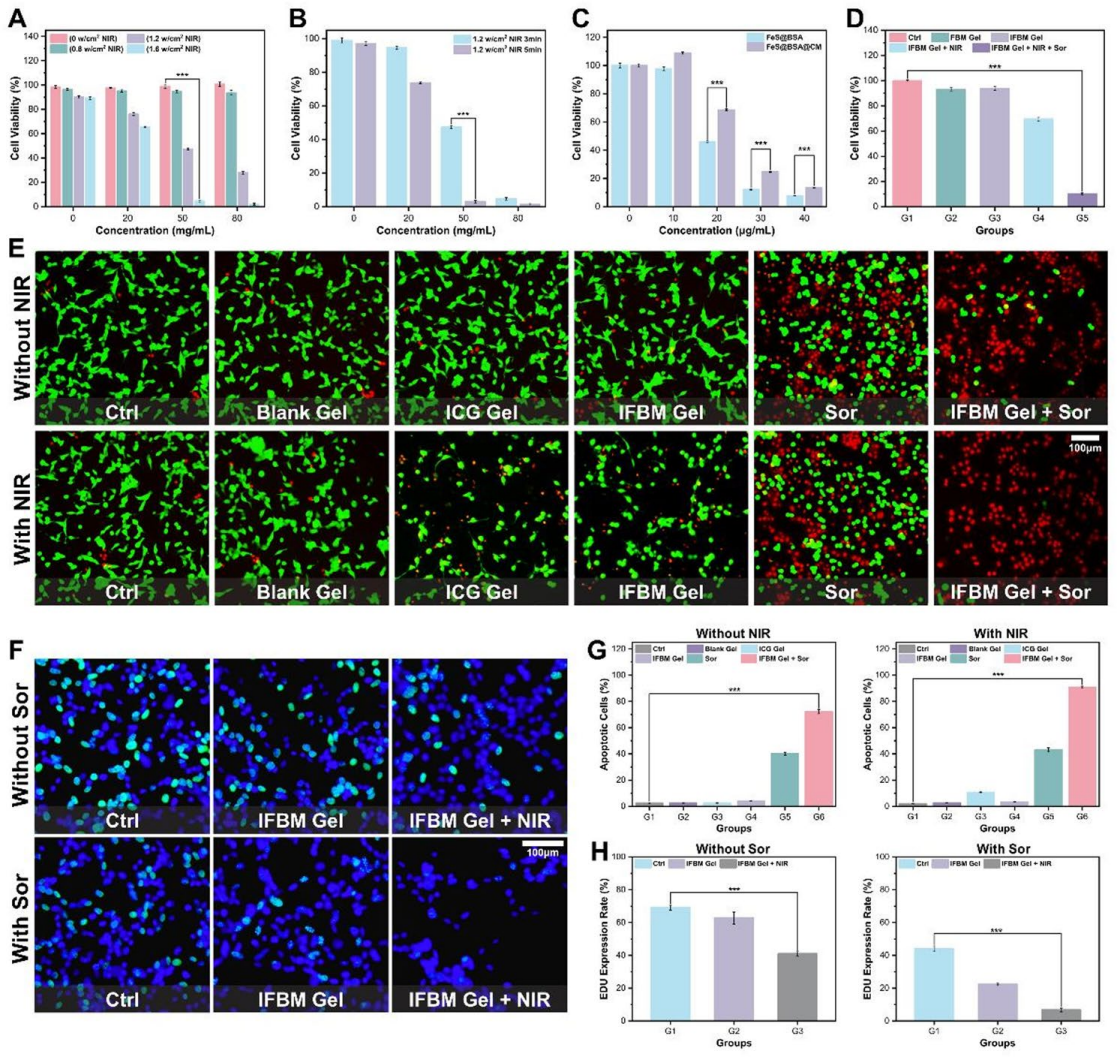
**A**) Effect of different concentrations of ICG gel on CT26 cell survival under NIR irradiation with different powers (*n* = 4, mean ± SD). **B**) Effect of different time of 1.2 W/cm2 NIR irradiation on the survival rate of CT26 cells (*n* = 4, mean ± SD). **C**) Effects of different concentrations of FeS@BSA(@CM) on CT26 cell survival (*n* = 4, mean ± SD). **D**) Effects of different groups of hydrogels on CT26 cell survival (G1: Ctrl group, G2: FBM gel group, G3: IFBM gel group, G4: IFBM gel + NIR group, G5: INS group) (*n* = 4, mean ± SD). **E**) Live-dead staining photos of CT26 cells in different groups, **G**) corresponding quantitative analysis (G1: Ctrl group, G2: Blank gel group, G3: ICG gel group, G4: IFBM gel group, G5: Sor group, G6: IFBM gel + Sor group) (*n* = 3, mean ± SD). **F**) EDU staining analysis of different groups of CT26 cells, **H**) corresponding quantitative analysis (G1: Ctrl group, G2: IFBM gel group, G3: IFBM gel + NIR group) (*n* = 3, mean ± SD). *p < 0.05, **p < 0.01, ***p < 0.001, and ****p < 0.0001

In this section, we conducted a series of in vitro cytotoxicity assays. Fig S5 depicts a schematic of near-infrared (NIR) irradiation. Figure 2A-B illustrate the effect of the gel’s photothermal capability on cell viability. The results revealed that increasing either the ICG concentration, the NIR power density, or the irradiation time progressively reduced cell viability, resulting in near‑complete cell ablation under the highest parameters. Because photothermal therapy is temperature‑dependent, these results were consistent with the heating profiles described above. We evaluated the influence of cell membrane (CM) coating by comparing FB NCs with FBM NCs. Figure 2C indicates that the CM coating moderately attenuated cytotoxicity, presumably because the membrane delays FB NC release and therefore slows ferroptosis. Subsequently, this study incubated CT26 cells with Sor to investigate its cytotoxicity (Fig. S6), and cell viability decreased progressively with increasing Sor concentration; however, approximately 20% of cells remained viable even at the highest dose, highlighting the limited efficacy of Sor monotherapy against colon cancer, a finding consistent with its current non‑use in this indication. Afterward, the effect of the combined treatment (IFBM gel + NIR + Sor) on cells was subsequently investigated. As illustrated in Fig. 2D, the combined treatment reduced cell viability to ~ 10%, which was markedly lower than either IFBM gel + NIR alone (~ 75%) or Sor alone (~ 40%), demonstrating a significant synergistic effect. To further evaluate the effects of the various treatments on CT26 cells, live/dead staining was conducted (Fig. 2E), and the associated quantitative analysis is illustrated in Fig. 2G. Neither NIR irradiation alone nor exposure to blank gel affected cell viability, whereas photothermal treatment or Sor monotherapy resulted in scattered red‑stained dead cells. However, almost all cells were non‑viable in the IFBM gel + NIR + Sor (INS) group. To examine the effects of each regimen on cell proliferation, EdU assays were conducted. Figure 2F depicts the results, with the corresponding quantitative analysis presented in Fig. 2H. Control cells proliferated normally, but cells treated with IFBM gel + NIR or with Sor alone exhibited inhibited proliferation, yet ~ 40% of the population remained in cycle. In contrast, almost no cells in the INS group transitioned into the proliferative phase, highlighting the enhanced antiproliferative synergy of the combined therapy.

### In vivo antitumor efficacy and biocompatibility of the INS strategy

**Fig. 3:**
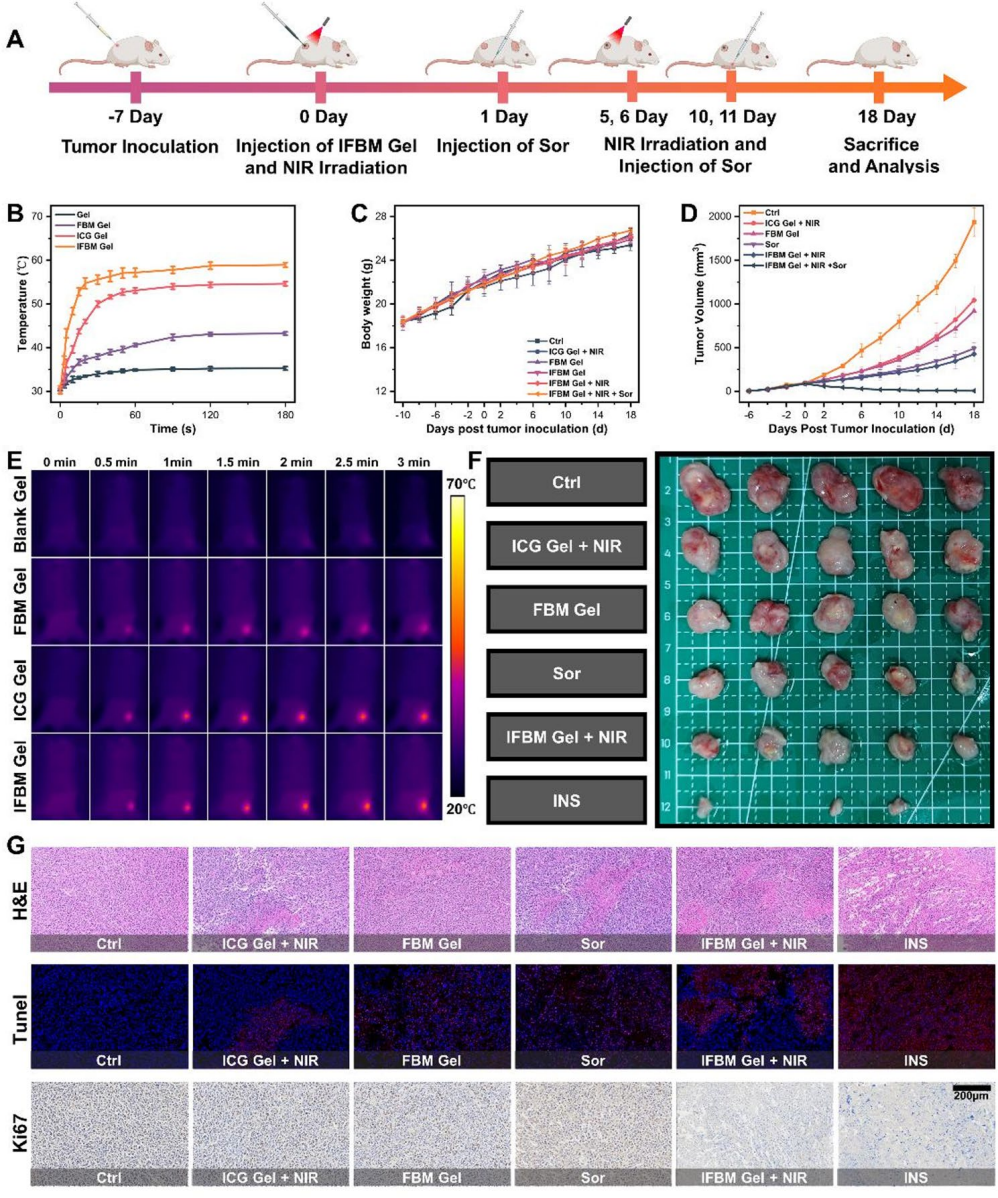
**A**) Flow chart of in vivo experiments. **B**) In vivo temperature-time change curves of different groups of gel under 1.2 W/cm2 NIR irradiation, and **E**) corresponding thermal imaging pictures (*n* = 3, mean ± SD). **C**) Body weight-time change curves of different groups of mice (*n* = 5, mean ± SD). **D**) Tumor volume-time change curves of different groups of mice (*n* = 5, mean ± SD), and **F**) corresponding tumor photographs. **G**) H&E, Tunel, and Ki67 analysis pictures of tumor sections in different groups of mice. *p < 0.05, **p < 0.01, ***p < 0.001, and ****p < 0.0001

In vivo experiments were subsequently conducted to evaluate the antitumor potential of the INS strategy. Figure 3A illustrates the experimental flowchart of the mouse studies. The photothermal performance of each gel formulation in mice was initially evaluated, and the temperature–time profiles depicted in Fig. 3B and the corresponding infrared thermal images are illustrated in Fig. 3E. NIR irradiation increased the intratumoral temperature to > 50 °C, indicating efficient photothermal conversion that was minimally impeded by the skin barrier. The relevant parameters of the mice were subsequently recorded during the experimental process. As illustrated in Fig. 3C, the body-weight trajectories did not differ significantly among the treatment groups. Tumor volume changes are presented in Fig. 3D, and representative tumor images are illustrated in Fig. 3F. Representative photographs of tumor-bearing mice on day 18 are provided in Fig. S7.

To examine the effects of different treatments on tumors at the tissue level, histological staining was performed on tumor sections from each group (Fig. 3G). Hematoxylin and eosin (H&E) staining revealed minimal apoptosis or necrosis in the control (Ctrl) group, whereas all treated groups exhibited varying degrees of tumor cell necrosis. The INS group exhibited the most extensive disruption of tumor architecture. Consistently, terminal deoxynucleotidyl transferase dUTP nick end labeling (TUNEL) staining revealed predominantly viable cells with blue fluorescence in the Ctrl tumors, whereas necrotic cells (red fluorescent) increased across treatment groups and were most abundant in the INS group, consistent with H&E results. In the Ki‑67 assay, nuclei in the Ctrl group stained brown, indicating active proliferation. Photothermal treatment alone or combined with FBM gel produced only mild growth inhibition, and Sor monotherapy yielded a modest antiproliferative effect without overt cytotoxicity. In contrast, the IFBM gel + NIR (IN) group both suppressed proliferation and partially disrupted tumor architecture, whereas the INS group exhibited almost no Ki‑67‑positive nuclei and abundant residual cell debris, highlighting its potent antitumor activity. Major organs were subsequently examined using H&E staining (Figs. S8A–F). No discernible structural or histopathological abnormalities were observed in any treated group compared with the Ctrl group, indicating negligible systemic toxicity and confirming the favorable biocompatibility of IFBM gel.

### RNA-seq, mIF, and flow cytometry reveal that the INS strategy leads to immune activation

**Fig. 4:**
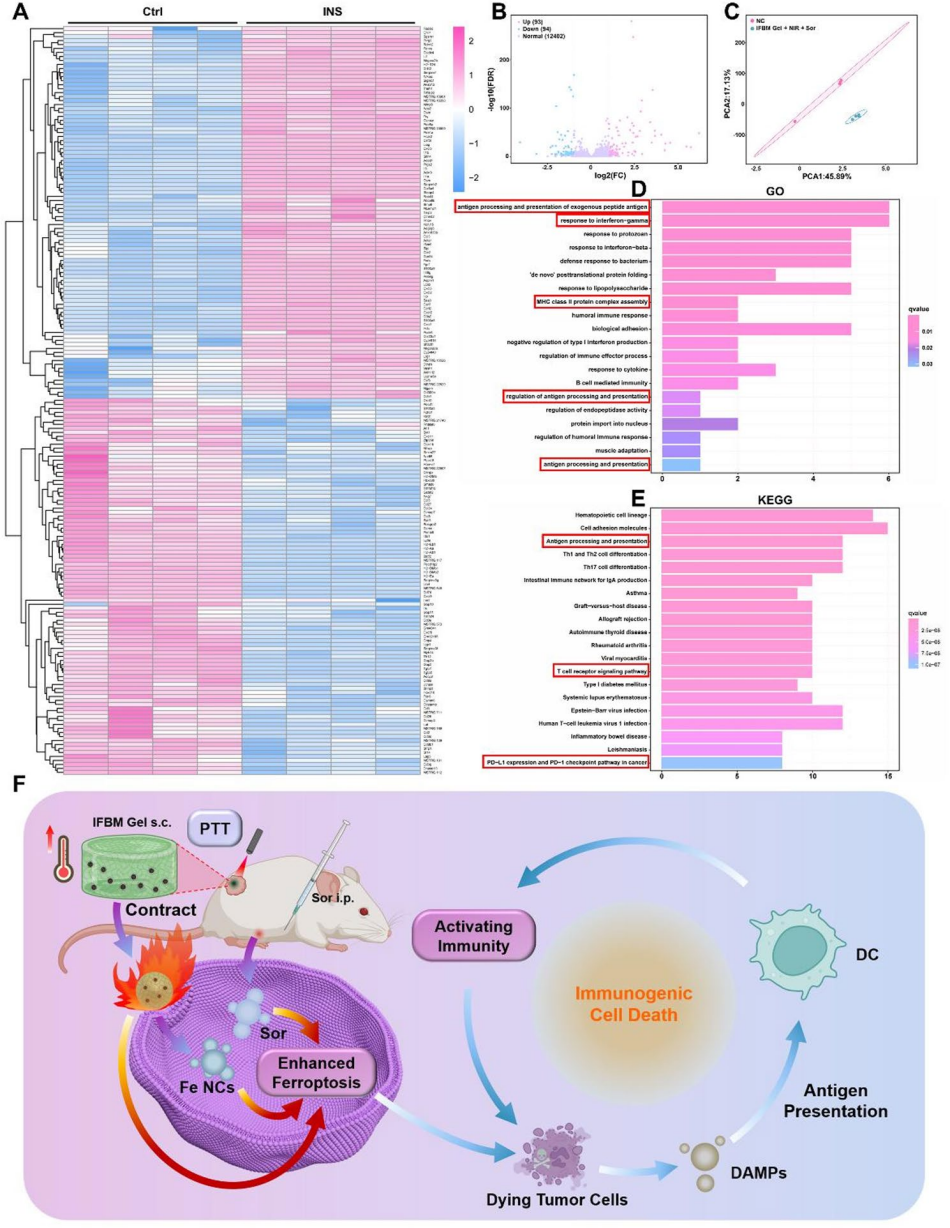
**A**) Heat map of differentially expressed gene expression in different groups. **B**) Volcano plot of gene expression differences. **C**) Principal component analysis plot of different groups. **D**) Histogram of GO pathway enrichment of differentially expressed genes in different groups (biological process). **E**) Histogram of KEGG pathway enrichment of differentially expressed genes in different groups. **F**) Conjecture diagram of antitumor mechanism of INS combination therapy (Created with bioRender.com)

Eukaryotic transcriptome sequencing was performed on tumor tissues to clarify the underlying pathway through which IFBM gel modulates the antitumor immune response. The heatmap (Fig. 4A) demonstrates a significant difference in gene expression between Ctrl and INS groups. The volcano plot (Fig. 4B) revealed 93 upregulated genes and 94 downregulated genes with a false discovery rate (FDR) > 1 and |log_2_(FC)| > 1, highlighting the significant transcriptional changes induced by the INS strategy. Additionally, the principal component analysis plot (Fig. 4C) illustrated distinct separation between Ctrl and INS groups, with the INS group exhibiting lower variability, indicating the stable effects of the treatment.

Gene ontology (GO) analysis of the biological process was subsequently performed (Fig. 4D). The analysis revealed that the expression of ‘antigen processing and presentation of exogenous peptide antigen,’ ‘response to interferon-gamma,’ ‘MHC class II protein complex assembly, ' ‘regulation of antigen processing and presentation,’ and ‘antigen processing and presentation’ in the tumor tissues of the INS group was significantly higher than that of the Ctrl group, suggesting that IFBM gel enhances the body’s antitumor immunity through antigen presentation. Figure 4E illustrates the Kyoto encyclopedia of genes and genomes (KEGG) pathway enrichment analysis for differentially expressed genes, revealing that the expression of ‘antigen processing and presentation,’ ‘T cell receptor signaling pathway,’ and ‘PD-L1 expression and PD-1 checkpoint pathway in cancer’ was significantly increased in the INS group compared with the control group, highlighting their relevance to antigen presentation and tumor immunity. Therefore, it can be deduced that IFBM gel stimulated the body’s antitumor immunity through antigen presentation to eradicate the tumor. The proposed mechanism is illustrated in (Fig. 4F) and will be further validated in subsequent experiments.

**Fig. 5:**
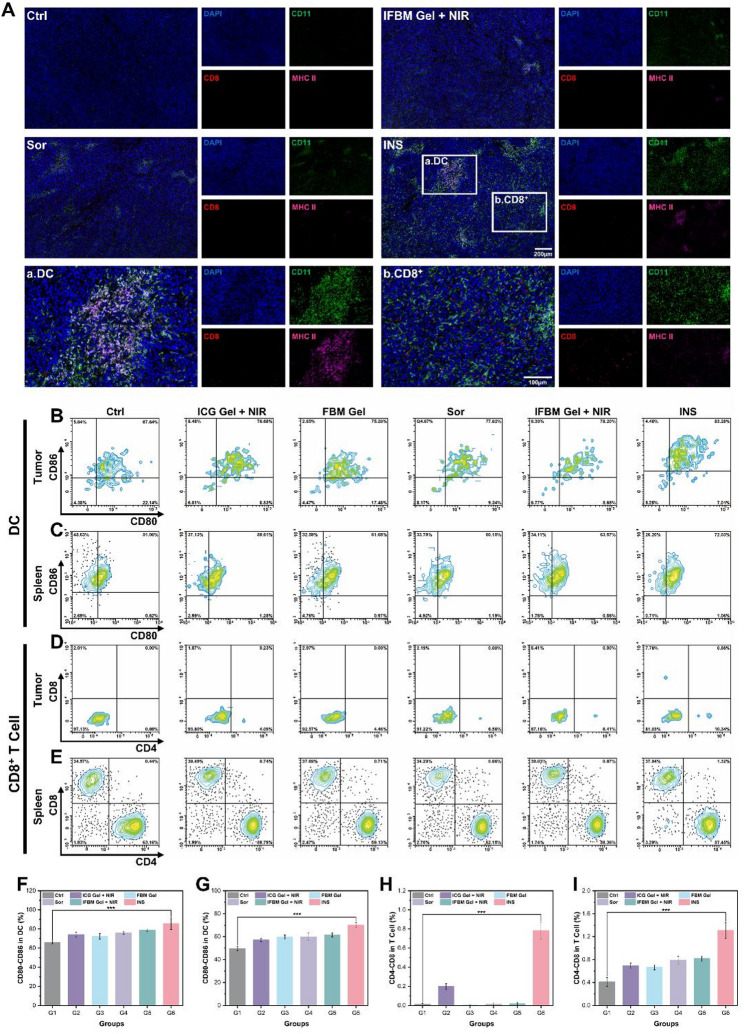
**A**) mIHC staining plots of tumor sections from different groups of mice (blue: DAPI, green: CD11, red: CD8, pink: MHC2). DC flow cytometry plots of different groups of mouse tumors **B**) & spleen **C**), **F**) and **G**) corresponding quantitative plots, T cell flow cytometry plots of different groups of mouse tumors **D**) & spleen **E**), **H**) and **I**) corresponding quantitative plots (G1: Ctrl group, G2: ICG gel + NIR group, G3: FBM gel group, G4: Sor group, G5: IFBM gel + NIR group, G6: INS group). Data are presented as mean ± SD (*n* = 3). *p < 0.05, **p < 0.01, ***p < 0.001, and ****p < 0.0001

To clarify the antitumor immune response, multiplex immunohistochemistry (mIHC) staining analysis was conducted on tumor sections from various groups (Fig. 5A). The results revealed minimal CD11 green fluorescence in the Ctrl group, signifying minimal immune cell infiltration. Conversely, tumor tissues from IN and Sor groups exhibited increased green fluorescence signals, along with a small amount of major histocompatibility complex class II (MHC II) pink fluorescence, indicating that both treatments exerted tumor-killing effects and enhanced the body’s immune response. In the INS group, CD11 and MHC II signals were significantly increased, and red fluorescence from CD8 was observed in some areas, further confirming that the combination therapy enhanced the infiltration of DCs and CD8^+^ T cells, thereby effectively activating antitumor immunity *in vivo*.

To further validate the immune-activating efficacy of this strategy, DCs in the tumor tissues of mice from various groups were analyzed using flow cytometry (Fig. 5B) and quantitatively assessed (Fig. 5F). The findings revealed that CD80 and CD86 expression in the Ctrl group was 67.64%, whereas various treatments induced DC cell infiltration, with the INS group demonstrating a significantly higher proportion at 83.28% compared to the Ctrl group, suggesting that the INS strategy could significantly promote DC cell maturation. Figure 5C illustrates the flow cytometry analysis of DCs in the spleens of mice, whereas Fig. 5G presents the corresponding quantitative data. The INS group exhibited a significantly higher percentage of DCs (72.03%) compared to the Ctrl group (51.06%), further substantiating that the INS strategy facilitated DC maturation throughout the body, thereby enhancing the systemic antitumor immune response. Figure 5D-E depict the T cell flow cytometry of the tumor and spleen, respectively, whereas Figs. 5H and J provide their quantitative analysis. In the case of spleen cells, the proportion of CD4^+^ and CD8^+^ T cells in the INS group was 1.32%, whereas the Ctrl group was 1%, indicating that the INS strategy effectively enhanced T-cell infiltration. This further illustrated that the INS strategy effectively stimulated the *in vivo* immune response in mice.

Additionally, to further validate the long-term antitumor immune-activating efficacy induced by the INS strategy, CD3^+^ T cells as well as CD44^+^CD62L^−^ T cells in the spleens of mice from different groups were analyzed by flow cytometry. As shown in Fig. S9A and its corresponding Fig. S9C, the INS group exhibited a markedly higher proportion of CD3^+^ T cells compared with the Ctrl group, increasing from 14.90% to 42.29%. Meanwhile, the percentage of CD44^+^CD62L^−^ T cells (Fig. S9B and its corresponding Fig. S9D) was also significantly elevated in the INS group (36.68%) relative to the Ctrl group (10.25%). These results indicate that the INS strategy effectively promotes the generation of effector memory T cells, thereby activating long-term systemic antitumor immunity and highlighting its superior potential in suppressing tumor recurrence and metastasis.

### Inhibition of metastasis and recurrence further confirms the tumor immune activation of the INS strategy

**Fig. 6:**
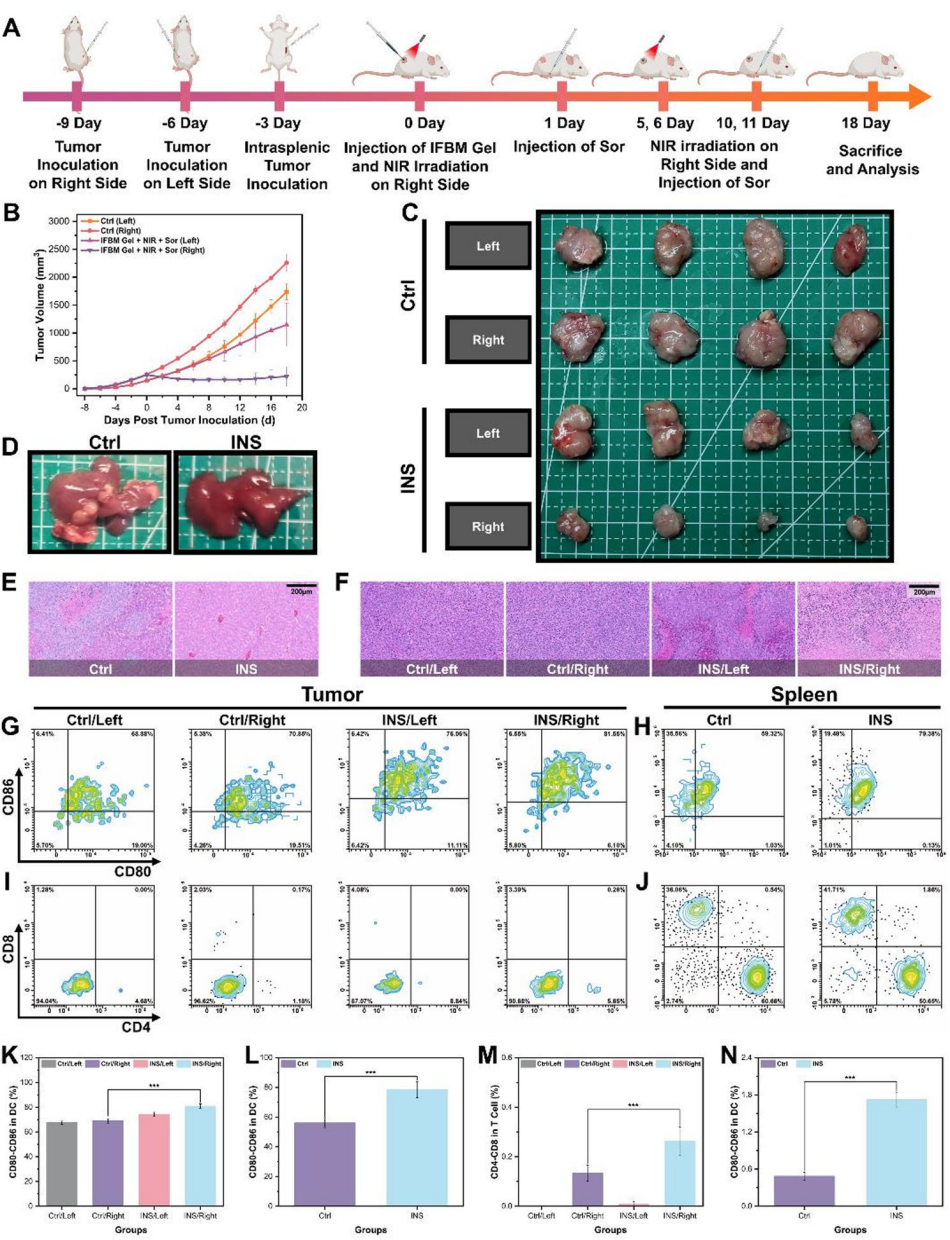
**A**) Flow chart of antitumor metastasis experiment in vivo. **B**) Tumor volume-time change curves of different groups of mice (*n* = 4, mean ± SD), **C**) photograph of corresponding mice tumors, and **F**) photographs of corresponding H&E staining. **D**) Photos of the livers of different groups of mice, **E**) corresponding H&E staining photographs. DC flow cytometry images of tumors **G**) & spleen **H**) of different groups of mice, **K**) and **L**) corresponding quantitative images (*n* = 3, mean ± SD). T cell flow cytometry images of different groups of mouse tumors **I**) & spleen **J**), and **M**) and **N**) corresponding quantitative images (*n* = 3, mean ± SD). *p < 0.05, **p < 0.01, ***p < 0.001, and ****p < 0.0001

Although INS strategy effectively induced local tumor suppression and activated the body’s antitumor immune response, its efficacy in preventing tumor recurrence and metastasis necessitated additional investigation. Bilateral tumor and liver metastasis mouse models were established for this investigation (Fig. 6A). Additionally, Figs. 6B-C illustrate the alterations in tumor volume and corresponding images of tumors from various groups of mice, respectively. The volume of the left-side tumors in the Ctrl group remained comparable to the volume of the right-side tumors three days earlier, suggesting that the growth of the right-side tumors exhibited minimal effect on the left-side tumors. Conversely, the right-side (treatment side) tumor volume was significantly reduced in the INS group, whereas the volume of the left-side tumors in the INS group decreased by approximately one-third relative to the left-side tumors in the Ctrl group. Figure 6D (liver images) and 6E (corresponding H&E staining) demonstrate that multiple tumor metastases were observed in the liver of the Ctrl group, whereas no significant tumor metastases were detected in the INS group. The results demonstrated that the INS strategy effectively activated tumor immunity and inhibited tumor recurrence and metastasis systematically. Figure 6F indicates the H&E staining results of bilateral tumor sections from mice in different groups. The bilateral tumors in the Ctrl group exhibited structural integrity, with no discernible cell necrosis or structural damage. In the INS group, significant structural degradation of the right-side tumors was observed, with a large number of necrotic tumor cells present. On the left side, partial structural destruction and necrotic areas were observed, further substantiating that the INS strategy effectively eradicated distant tumor metastatic foci by activating the body’s immune system.

Flow cytometry was subsequently performed on tumors and spleens from mice in different groups to examine their immune-activating potential. The results of DC and T cell flow cytometry are illustrated in Figs. 6G–J, respectively, with the corresponding quantitative analyses presented in Figs. 6K–N. The findings demonstrated that the DC and T cell levels in the bilateral tumors of the Ctrl group were similar, and both were lower than those in the left side (the untreated side) of the INS group, and significantly lower than those in the right side (the treated side). In addition, the DC and T cell content in the spleens of the Ctrl group was significantly lower than that in the INS group. Meanwhile, the proportions of CD3^+^ T cells and CD44^+^CD62L^−^ T cells in the spleens of mice in the INS group were significantly higher than those in the Ctrl group (Figs. S10A-D). These findings indicated that the INS strategy effectively promoted DC cell maturation and T-cell infiltration, further substantiating that this combined treatment effectively activated systemic immune responses and long-term antitumor immunity, thereby suppressing tumor recurrence and metastasis.

### Antitumor mechanism of the INS strategy

**Fig. 7:**
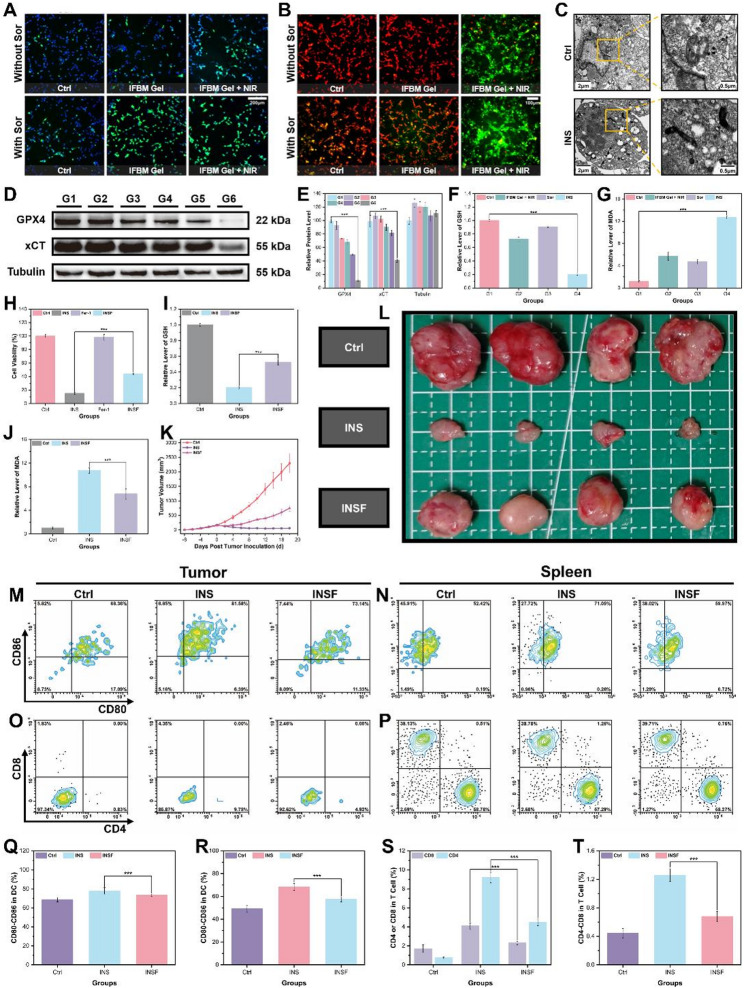
**A**) ROS content analysis of CT26 cells in different groups. **B**) JC1 staining analysis of CT26 cells in different groups. **C**) Transmission electron micrographs of mitochondria of CT26 cells in different groups. **D**) WB analysis of CT26 cells of different groups, **E**) corresponding quantitative graph (G1: Ctrl group, G2: ICG gel + NIR group, G3: FBM gel group, G4: Sor group, G5: IFBM gel + NIR group, G6: INS group) (*n* = 3, mean ± SD). **F**) & **G**) The quantitative graphs of GSH and MDA in different groups (G1: Ctrl group, G2: ICG gel + NIR group, G3: Sor group, G4: INS group) (*n* = 3, mean ± SD). **H**) CCK-8 analysis of CT26 cells after treatment in different groups (*n* = 4, mean ± SD). **I**) & **J**) The quantitative graphs of GSH and MDA after hydrogel treatment in different groups (*n* = 3, mean ± SD). **K**) The tumor volume-time change curve of mice in different groups, and **L**) photographs of corresponding tumors (*n* = 4, mean ± SD). DC flow cytometry plots of tumor **M**) & spleen **N**) in different groups of mice, **Q**) and **R**) corresponding quantitative plots (*n* = 3, mean ± SD). T cell flow cytometry plots of tumor **O**) & spleen **P**) in different groups of mice, S) and T) corresponding quantitative plots (*n* = 3, mean ± SD). *p < 0.05, **p < 0.01, ***p < 0.001, and ****p < 0.0001

To clarify the tumor-suppressing mechanism of the multi-therapy synergy, reactive oxygen species (ROS) assays were conducted on CT26 cells from various subgroups (Fig. 7A), with the associated quantitative results presented in Fig. S11A. A minimal concentration of green‑fluorescent ROS was observed in the Ctrl group. Treatment with IFBM gel or Sor increased ROS levels compared to the Ctrl group, whereas approximately 80% of the cells in the INS group generated ROS, thereby confirming that this multi‑therapy synergy induced cell death through oxidative stress. To validate these findings, JC-1 assays were conducted (Fig. 7B), with the associated quantitative results displayed in Fig. S11B. In the Ctrl group, the mitochondrial membrane potential was stable and exhibited consistent red fluorescence. After Sor treatment, a minor fraction of cells exhibited oxidative stress, indicated by green fluorescence. However, cells in the INS group exhibited significantly stronger oxidative stress, attributed to photothermal heating that liberated iron ions from the gel and subsequently induced ferroptosis. Transmission electron microscopy further confirmed these findings (Fig. 7C). The mitochondria in the Ctrl group exhibited normal morphology, whereas those in the INS‑treated cells exhibited significant wrinkling, indicative of mitochondrial alterations associated with ferroptosis.

Ferroptosis-related proteins were examined using Western blot (WB) (Fig. 7D), and their expression levels were quantified (Fig. 7E). The findings demonstrated modified protein profiles across the various groups: GPX4 and xCT were significantly down‑regulated in the INS group, suggesting that the INS strategy inhibited tumors by inducing cellular ferroptosis. Glutathione (GSH) and malondialdehyde (MDA), two key biochemical markers of ferroptosis, were subsequently measured. As illustrated in Fig. 7F, GSH levels were decreased in all treated cells, with the INS group maintaining only 20% of the GSH content observed in the Ctrl group. Conversely, Fig. 7G illustrates that MDA levels increased in all treatment groups; in the INS group, MDA increased nearly 12‑fold compared to the Ctrl group. These findings further validated the occurrence of ferroptosis by the INS strategy and demonstrated that Sor effectively induced and enhanced ferroptosis.

To further confirm the role of ferroptosis in tumor suppression, Ferrostain-1 (Fer-1), a ferroptosis inhibitor, was introduced into the experiments. Figure 7H depicts the results of the *in vitro* Cell Counting Kit-8 (CCK-8) assays. Fer-1 alone had minimal impact on cell viability; however, the IFBM gel + NIR + Sor + Fer-1 (INSF) group demonstrated a 30% enhancement in cell activity compared to the INS group, indicating that Fer-1 mitigated INS-induced ferroptosis. Moreover, GSH and MDA assays were conducted, as depicted in Figs. 7I-J. The GSH level increased, whereas the MDA levels decreased in the INSF group compared to the INS group, indicating that Fer-1 partially mitigated the ferroptosis-inducing effect of the INS strategy. These findings further corroborated that the INS strategy could elicit cell death via ferroptosis.

Considering that inhibiting ferroptosis may reduce ICD, potentially leading to tumor immunosuppression and decreased antitumor effects, this hypothesis was further validated through experiments conducted on mice, as illustrated in Fig. S12. Additionally, Fig. 7K depicts the changes in tumor volume across different groups, whereas Fig. 7L illustrates the tumor images. The tumor volume in the INSF group was significantly greater than that in the INS group, thereby confirming the critical role of ferroptosis in the *in vivo* antitumor process. Fig. S13 displays the H&E staining analysis of tumor sections from various groups. The findings demonstrated significant destruction of the tumor architecture in the INS group, with numerous necrotic tumor cells observed. However, the INSF group demonstrated only partial structural destruction, suggesting that Fer-1 partially mitigated the tumor-suppressing efficacy of the INS strategy.

**Scheme. 1:**
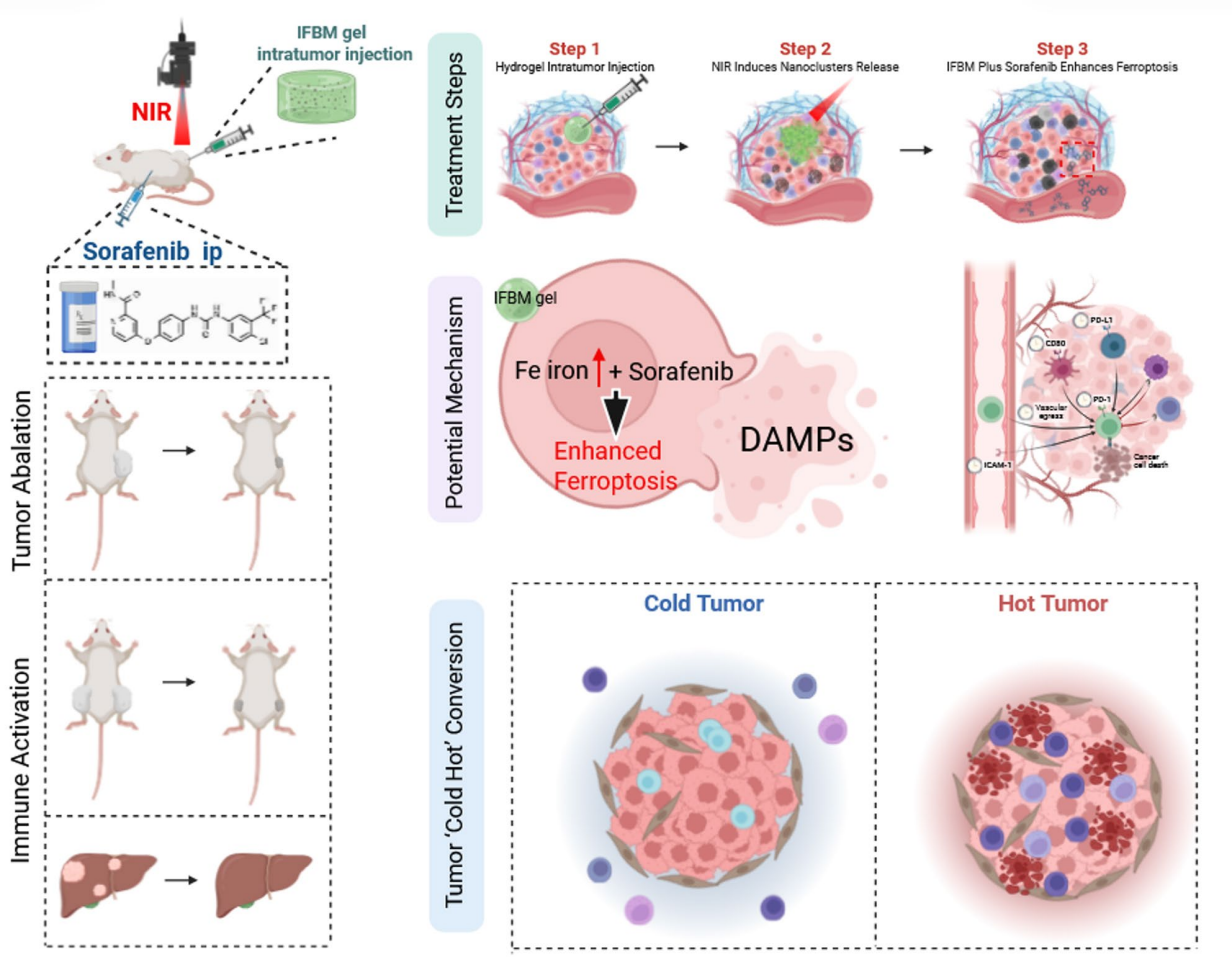
Combined antitumor mechanisms of INS Strategy (Created with bioRender.com).

Subsequently, flow cytometry assays were conducted on tumors and spleens from the different groups. Figure 7M-N depict the DC flow cytometry, whereas Figs. 7O-P illustrate the T cell flow cytometry, and Figs. 7Q–T present their corresponding quantitative analysis. In the INSF group, the quantity of mature DC cells and the extent of T-cell infiltration in the tumor and spleen were diminished compared to the INS group. Moreover, the proportions of CD3^+^ and CD44^+^CD62L^−^ T cells in the INSF group were reduced compared with those in the INS group (Figs. S14A-D). This pertained to the partial inhibition of ferroptosis, which subsequently diminished ICD and impeded the activation of robust antitumor immunity. The tumor-suppressing mechanism of the INS strategy was summarized in the provided Scheme 1.

## Discussion and conclusion

This study engineered a thermosensitive IFBM hydrogel system that integrates FBM NCs and ICG, which, when combined with Sor, induces PTT and ferroptosis in colorectal cancer models. The system exhibited high photothermal conversion efficiency, attaining temperatures exceeding 60 °C under NIR irradiation, facilitating controlled degradation and on-demand release of iron ions with an approximate cumulative release of 80% over five cycles. *In vitro* assays revealed significant cytotoxicity in CT26 cells, marked by increased ROS levels, GSH depletion, MDA accumulation, and collapse of mitochondrial membrane potential, resulting in near-complete cell death under combined treatment. *In vivo*, intratumoral injection combined with NIR irradiation and Sor administration markedly suppressed primary tumor growth, enhanced DC maturation (increased CD80/CD86 expression), and increased CD8^+^ T-cell infiltration, resulting in the effective suppression of distant liver metastases without detectable systemic toxicity, as verified by histological examination of major organs.

These findings highlight the complex interplay between ferroptosis and ICD, where ferroptosis acts as a significant catalyst for ICD by facilitating the release of DAMPs, thus activating antitumor immunity [19]. Ferroptosis, characterized by iron-dependent lipid peroxidation and differentiated from apoptosis or necroptosis, enhances ICD through unrestricted accumulation of lipid peroxides, which undermine membrane integrity and expose immunogenic signals [20]. Our experiments demonstrated that the combination of Sor-mediated GPX4 inhibition and iron supplied by FBM exacerbated lipid peroxidation, as indicated by a significant increase in MDA levels in treated cells compared to controls. This corresponds with recent findings indicating that ferroptosis-induced lipid metabolites, act as danger signals that attract and mature DCs, thereby promoting a pro-inflammatory TME [21]. The interaction between ferroptosis and ICD is also reflected in the manner in which ROS alters the TME. In ferroptotic cells, whereas ROS assays revealed an increase in oxidative stress that likely directly killed tumor cells and enhanced the TME’s susceptibility to immune infiltration, as mIHC revealed increased CD11c^+^ DCs and MHC II expression in treated tumors [22]. In contrast to conventional apoptosis, which frequently induces tolerogenic responses, ferroptosis-mediated ICD in our model stimulated a powerful antitumor immunity, as transcriptome sequencing of tumor tissues revealed elevated IFN-γ pathways and increased MHC II expression, thereby facilitating antigen presentation and T-cell activation [23]. In our bilateral tumor model, this activation was reflected in the suppression of contralateral tumors, with flow cytometry revealing increased splenic CD8^+^ T-cell proportions, indicating systemic immune activation similar to the effects of vaccination [24].

Based on these observations, we propose consolidating our findings into an innovative conceptual framework: the “tumor in situ vaccine repository” (TISVR). The TISVR envisions the IFBM hydrogel as a localized, dynamic antigen reservoir that utilizes ferroptosis-induced ICD to maintain an endogenous antigen reservoir at the tumor site. In contrast to conventional vaccines that depend on predefined exogenous antigens, which frequently exhibit inadequate immunogenicity and off-target effects, the TISVR utilizes the tumor’s intrinsic neoantigens released during ferroptotic death, thereby establishing a self-sustaining immune loop [25]. In our system, NIR-triggered PTT liquefies the hydrogel (> 43 ℃), releasing FBM NCs to catalyze iron-dependent lipid peroxidation, whereas Sor enhances ferroptosis, facilitating successive waves of ICD. This replicates booster vaccinations, as demonstrated by the multi-cycle photothermal stability and sustained iron ions release, which preserved antitumor efficacy over 18 days *in vivo *[26]. The vaccine-like efficacy of TISVR was further corroborated by the ablation of liver metastases, with transcriptome analysis indicating enriched pathways for effector memory T-cell activation and IFN-γ signaling, suggesting long-term memory immunity [27].

The TISVR framework transcends solely direct cytotoxicity by tackling TME immunosuppression, a major barrier in cancer therapy. Ferroptosis in tumor cells disrupts lipid homeostasis, releasing oxidized lipids that inhibit myeloid-derived suppressor cells, thus mitigating immune evasion [28]. Our data align with this, as treated tumors exhibited reduced immunosuppressive markers and improved DC maturation, fostering a “hot” TME conducive for CD8^+^ T-cell infiltration. Furthermore, the integration of PTT in TISVR intensifies this effect; hyperthermia accelerates hydrogel degradation and triggers heat shock proteins that function as additional DAMPs, synergizing with ferroptotic lipids to enhance antigen cross-presentation [29]. Our combined approach, in contrast to monotherapy, reduced tumor volume by over 80%, highlighting TISVR’s potential in overcoming resistance to single-agent treatment [30].

Recent advancements validate the TISVR’s potential in refractory cancers. In pancreatic ductal adenocarcinoma, where mesenchymal states provide resistance to ferroptosis, nanoparticle-mediated iron delivery similar to our NCs has induced ICD and enhanced tumor sensitivity to checkpoint inhibitors [31]. In HCC, ferroptosis can promote the transformation of M2 macrophages into M1 macrophages, hereby reshaping the TME, consistent with our mIHC data demonstrating increased T-cell infiltration [32]. The TISVR achieves this by establishing sustained antigen release, in contrast to the temporary ICD blockade from individual agents, which may enhance response rates in “cold” tumors, such as those in our CT26 model [33]. Furthermore, in lung cancer models, ferroptosis has been associated with enhanced PD-L1 degradation, complementing immunotherapy; our system’s capacity to modulate IFN-γ pathways indicates TISVR could enhance PD-1 blockade [34].

The expansion of the TISVR concept reveals that ferroptosis’s intersection with other regulated cell deaths (RCDs) broadens its vaccine-like potential. For instance, ferroptosis can interact with pyroptosis through gasdermin pores that facilitate lipid peroxide efflux, thereby enhancing DAMP release in our heated hydrogel environment [35]. *In vivo*, this synergy probably contributed to the observed TUNEL-positive apoptosis in tumors, integrating ICD modalities for enhanced antigen diversity [36]. In our model, ROS and JC-1 assays demonstrated the accumulation of excessive ROS, resulting in mitochondrial damage and ultimately ferroptotic rupture [37]. This designates TISVR as a multi-RCD inducer, overcoming single-pathway resistance common in heterogeneous tumors [38].

Clinically, TISVR addresses deficiencies in existing in situ vaccines (ISVs), which frequently depend on radiotherapy or oncolytic but lack the integration of controlled ferroptosis. Recent trials of ISVs in melanoma have demonstrated a response rate of 20%–30%, limited by inadequate antigen persistence; our repeated NIR cycles enhance this effect, simulating multi-dose vaccines [39]. In glioma, ferroptosis agonists exhibit improved bblood-brain barrier penetration, indicating TISVR’s applicability to invasive cancers via injectable hydrogels [40].

However, challenges persist: NIR penetration limits deep tumors; however, fiber-optic modifications may alleviate this limitation [41]. Variability in ferroptosis susceptibility among patients, influenced by SLC7A11 polymorphisms, necessitates the use of biomarkers such as GPX4 expression for personalized treatment [42]. Additionally, therapies that require endogenous substrates to mediate tumor suppression, such as ferroptosis-based approaches and chemodynamic therapy, may experience a decline in efficacy once those TME’s substrates are depleted. Therefore, TISVR may incorporate supplemental exogenous substrate sources to provide sustained tumor suppression through rational design [43]. Simultaneously, local injection of TISVR inevitably causes tumor damage. While this damage is significantly reduced compared to surgical treatment, it still increases the risk of tumor cells entering the systemic circulation [44]. For patients with immunodeficiency or tumors difficult to precisely locate, TISVR may struggle to function effectively due to challenges in local tumor injection or the inability to activate systemic antitumor immunity [45].

Future directions for TISVR include integrating CRISPR-edited NCs to target ferroptosis inhibitors, thereby enhancing ICD in resistant subtypes [46]. Combined strategies utilizing CAR-T cells may exploit TISVR’s antigen repository for prolonged chimeric antigen recognition [47]. TISVR demonstrates broad application potential by synergizing with any strategy that effectively activates endogenous antitumor immunity through rational design.

Our synergistic strategy exemplifies the TISVR, utilizing the synergy of ferroptosis and ICD to create a dynamic antigen reservoir that restructures the TME and induces vaccine-like immunity. This approach ablates primary tumors and metastases and offers sustained responses, providing a viable strategy for cancer treatment.

## Experimental section

### Materials, reagents, and instruments

FeCl_2_, Na_2_S-9H_2_O were procured from Chenyu Biotechnology Co., Ltd. (Hangzhou, China). BSA was obtained from BioSmile Biotechnology Co., PLGA-PEG-PLGA pristine gels were acquired from Daigang Biomaterial Co., and ICG was sourced from Acmec Biochemical Technology Co., Ltd. (Shanghai, China).

The MDA content assay kit was supplied by Solarbio (Beijing, China). GSH, GSSG, and the JC1 assay kit were provided by Beyotime (Shanghai, China). Reagents for flow cytometry, such as mouse FcR sealer, cell staining buffer, and erythrocyte lysate, were provided by Elabsceince (Wuhan, China). Ferrostatin-1 reagent was provided by MedChemExpress (Shanghai, China).

Rheological properties were determined by a rheometer (Haake Mars40, Germany). Absorption wavelength was determined by a UV-visible spectrophotometer (Jinghua Technology Instrument Co., Ltd, Shanghai, China). NIR was emitted by an 808 nm laser (Blueprint Optoelectronics Technology Co., Ltd, Beijing, China). Thermograms were taken through a thermal imaging lens (Guide Infrared Co., Ltd., Wuhan, China). Optical Density (OD) was determined by BioTeck Enzymometer (BioTeck Enzymometer, USA). Cellular optical photographs were observed and taken by an Olympus BX60 fluorescence microscope (Olympus, Germany). Mitochondrial structures were observed and photographed by a JEM1400 transmission electron microscope (JEOL, Japan). Tumor and tissue sections were observed and photographed using a Leica vertical microscope (DM6B, Leica, Germany).

All experiments were conducted at room temperature, unless otherwise specified.

### Solution preparation

ICG Solution Preparation: 2 mg of ICG powder was dissolved in 10 mL of deionized water to obtain a 200 µg/mL solution; stored at 4 °C for subsequent use.

Sor Solution Preparation: Commercially obtained Sor was diluted to prepare a 10 M stock solution, which was then further diluted with deionized water to obtain a working solution of 10 mM. The solution was stored at 4 °C for subsequent use.

Ferrostatin-1 Solution Preparation: Commercially obtained Ferrostatin-1 was diluted to 2 mM, and a working solution of 50 µg/mL was subsequently prepared. The solution was stored at 4 °C for subsequent use.

### Preparation of FB NCs and FBM NCs

FB NCs Preparation: 50.0 mg of BSA was dissolved in 6.0 mL of deionized water. Subsequently, 1.0 mL of FeCl₂ solution (37 mM) and 1.5 mL of Na₂S solution (37 mM) were added successively under vigorous stirring to ensure thorough mixing. The mixture was allowed to react at 4 °C for 12 h with shaking. The resultant solution was dialyzed (molecular weight cutoff: 8000–14000) against deionized water for 12 h at 4 °C to obtain a suspension of FB NCs. The suspension was subsequently diluted with deionized water to a final concentration of 1 mg/mL, frozen, and stored for subsequent use.

Cell Membrane (CM) Extraction: Cultured CT26 cells were harvested by digestion and subjected to lyophilization using deionized water at room temperature and at − 80 °C for 10 cycles. The cells were centrifuged at 1000 rpm to collect the supernatant, followed by centrifugation at 10,000 rpm to obtain the precipitate containing the cell membrane. FB NCs were subsequently mixed with an equal mass of cell membrane suspension in an ultrasonic shaker (100 W, 42 kHz) for 10 min. The mixture was centrifuged at 10,000 rpm for 5 min at 4 °C, and the precipitate was collected and resuspended in deionized water to formulate a 1 mg/mL suspension. This suspension was frozen and stored, resulting in FBM NCs. FB NCs and FBM NCs concentrations were calculated based on the actual Fe concentration (1 mg/mL of iron ions in 1 mg/mL of FBM NCs).

### Preparation of different components of gel

Preparation of PPP gel: Commercially available PLGA-PEG-PLGA original gel was mixed with deionized water at a 1:2 ratio. The solution was further diluted as needed, with a 1:1 ratio of concentrated PPP gel to deionized water to obtain the final working gel.

Preparation of Blank PPP gel: We mixed 1 mL of concentrated PPP gel with 1 mL of deionized water to obtain blank PPP (blank) gel, which was stored at 4 °C for subsequent use.

Preparation of ICG gel: We mixed 1 mL of concentrated PPP gel with 0.2, 0.5, or 0.8 mL of ICG solution, respectively, and deionized water was added to make the final volume of 2 mL. The solution was mixed thoroughly to obtain ICG gel with ICG concentrations of 20, 50, and 80 µg/mL, which were stored at 4 °C for subsequent use.

Preparation of FB gel or FBM gel: We mixed 1 mL of concentrated PPP gel with 0.2 mL of FB NCs or FBM NCs suspension (1 mg/mL), and deionized water was added to make a final volume of 2 mL. The solution was mixed thoroughly to obtain FB gel or FBM gel, which was stored at 4 °C for subsequent use.

Preparation of IFB gel or IFBM gel: We mixed 1 mL of concentrated PPP gel with 0.2 mL of FB NCs or FBM NCs suspension (1 mg/mL) and added 0.5 mL of ICG solution. Deionized water was added to make the final volume of 2 mL, and the solution was mixed thoroughly to obtain IFB gel or IFBM gel, which was stored at 4 °C for subsequent use.

### Appearance and temperature sensitivity testing of gels

We prepared 1 mL each of PPP gel, 50 µg/mL ICG gel, FBM gel, and IFBM gel at 4 °C and immediately photographed them to document their initial appearance. The samples were subsequently placed in a 37 °C water bath for 3 min, removed, and photographed again. Subsequently, the samples were placed in a 60 °C water bath for 3 min, and their appearance was recorded.

### Characterization of gels

The microstructure of FB NCs and FBM NCs was examined using transmission electron microscopy (*n* = 3). PPP gel morphology was assessed using scanning electron microscopy (*n* = 3). Rheological properties of the PPP gel were analyzed using modulus-temperature scanning from 4 to 60 °C. For spectroscopic analysis, 0.3 mL of PPP, ICG, FBM, and IFBM gels were mixed with 2.7 mL of deionized water, homogenized, and analyzed using a spectrophotometer over the 250–850 nm wavelength range (*n* = 3).

### Oxidation and degradation of NCs and gels

At 4 °C: We placed 1 mL of FBM gel and IFBM gel at 4 °C and sampled them at regular intervals for photographic documentation. In the preparation process, concentrated PPP gel was replaced with deionized water to obtain the corresponding diluted suspensions of FBM NCs and IFBM NCs. Additionally, 1 mL of each NC was placed at 4 °C and sampled at regular intervals for photographic documentation.

At 37 °C: We placed 1 mL of FBM gel and IFBM gel in a 37 °C water bath and sampled them at regular intervals for photographic documentation. Subsequently, we placed 1 mL of the diluted FBM NCs and IFBM NCs suspensions in a 37 °C water bath and sampled them at regular intervals for photographic documentation.

Under Photothermal Conditions: Two 1 mL samples of IFBM gel were taken. One was photographed after 3 min under 1.2 W/cm^2^ NIR, and the other was photographed after 15 min under 1.2 W/cm^2^ NIR.

### *In vitro* photothermal performance of gels

The effect of NIR power on the photothermal performance of gels: A 50 µg/mL ICG gel was irradiated for 3 min using 0, 0.8, 1.2, and 1.6 W/cm^2^ NIR, respectively, and the temperature of the gel was measured at regular intervals (*n* = 3).

To clarify the effect of ICG concentration on the photothermal properties of the gel, ICG gel with ICG concentrations of 20, 50, and 80 µg/mL was irradiated with 1.2 W/cm^2^ NIR for 3 min, and its temperature was measured at regular intervals (*n* = 3).

PPP gel, FBM gel, 50 µg/mL of ICG gel, and IFBM gel were irradiated with 1.2 W/cm^2^ NIR for 3 min, and their temperatures were measured at regular intervals, with corresponding infrared images taken (*n* = 3).

Additionally, 50 µg/mL ICG gel was subjected to on-off illumination, irradiated with 1.2 W/cm^2^ NIR for 3 min, followed by cooling for 3 min. This cycle was repeated 5 times, and the temperature was measured at regular intervals to investigate its photothermal stability (*n* = 3).

ICG gel was placed at room temperature, and its temperature was measured at regular intervals. ICG gel was placed in a 37 °C water bath for 3 min and subsequently placed at room temperature, and the temperature was measured regularly. Subsequently, ICG gel was placed in a 37 °C water bath for 3 min and subsequently irradiated with 1.2 W/cm^2^ NIR for 3 min, and the temperature was measured at regular intervals (*n* = 3).

### Substance release properties of gels

FeCl_3_ was dissolved in deionized water to prepare solutions of varying concentrations (*n* = 3). Spectral scanning was performed to establish an absorbance-wavelength curve of Fe^3+^. The absorbance-concentration relationship was obtained by fitting the absorbance-concentration data of Fe^3+^ with 303 nm as the absorption peak.

Subsequently, IFB gel and IFBM gel were subjected to 5 cycles of on-off photothermal treatment, and 0.1 mL of the supernatant was aspirated after 3 min of irradiation and 3 min of cooling, respectively (*n* = 3). The supernatant was subsequently added to 2.9 mL of deionized water, and the absorbance at 303 nm was measured. After substituting the absorbance-concentration equation, the Fe^3+^ concentration of the supernatant was determined. By comparing this with the total Fe^3+^ concentration, the release concentration of Fe^3+^ was calculated, and the release proportion-time curve was plotted.

### Cell culture

Mouse colon cancer cells (CT26) were obtained from Warner Bio (Wuhan, China) and cultured in RPMI1640 medium (Gibco, Waltham, MA, USA) supplemented with 10% fetal bovine serum (FBS; Gibco), 100 IU/mL penicillin, and 100 µg/mL streptomycin at 37 °C with 5% CO₂. When the cell density of CT26 cells exceeded 90%, the cells were inoculated into 6-, 24-, or 96-well plates for subsequent cell experiments.

### Evaluation of the *in vitro* antitumor effect of gel

The cultured cells were initially inoculated into 24-well plates and incubated for 24 h. Upon reaching a cell density of approximately 60%, the cells were treated as outlined below, and the subsequent absorbance was measured and analyzed using CCK-8 according to the instructions.

The specific treatments were as follows:

Effect of NIR power and ICG gel concentration on antitumor (*n* = 4): Cells were treated with different power NIR irradiation and varying concentrations of ICG gel for 3 min and subsequently cultured for 24 h.

Effect of NIR irradiation time on antitumor (*n* = 4): Cells were treated with 1.2 W/cm^2^ NIR irradiation of 50 µg/mL ICG gel for 3–5 min and subsequently cultured for 24 h.

Effect of the presence or absence of cell membrane on the antitumor activity of FB NCs (*n* = 4): FB NCs and FBM NCs were added to the culture medium, with the concentrations of FB NCs and FBM NCs set at 10, 20, 30, and 40 µg/mL, respectively, and subsequently cultured for 24 h.

Assessment of antitumor efficacy of different concentrations of Sor (*n* = 4): We added 10 mM Sor solution to the medium to achieve final concentrations of 2, 4, 6, …, 18, and 20 µM, and subsequently cultured them for 24 h.

Assessment of antitumor effects of different treatments (*n* = 4): Group 1 was left untreated; group 2 was soaked in FBM gel in culture medium for 3 min; group 3 was soaked in IFBM gel in culture medium for 3 min; group 4 cells were treated by irradiating IFBM gel with 1.2 W/cm^2^ NIR for 3 min; group 5 cells were treated with 1.2 W/cm^2^ NIR irradiation of IFBM gel for 3 min followed by the addition of Sor to the medium to achieve a final concentration of 10 µM. Subsequently, the cultures of groups 1–5 cells were continued for 24 h.

### Live-dead staining experiments of cells

Initially, the cultured cells were inoculated and incubated in 24-well plates for 24 h. Upon reaching a cell density of approximately 60%, the cells were divided into six groups (*n* = 3): Groups 1–6, with and without NIR irradiation (1.2 W/cm^2^). The subsequent treatments were performed as follows: Group 1: Immersion/irradiation without gel; group 2: Immersion/irradiation using blank gel for 3 min; group 3: Immersion/irradiation using ICG gel for 3 min; group 4: Immersion/irradiation using IFBM gel for 3 min; group 5: Sor was added to the medium to achieve a concentration of 10 µM; group 6: Immersion/irradiation utilizing IFBM gel for 3 min, and Sor was added to the medium to achieve a concentration of 10 µM. The cultures were subsequently incubated for 24 h, after which they were processed using a live-dead staining kit according to the manufacturer’s instructions. The stained cells were examined and photographed using a fluorescence microscope, and the images were analyzed using ImageJ software for image analysis.

### EDU, ROS, and JC1 analysis of cells

The cultured cells were initially seeded in 24-well plates and incubated for 24 h. Upon reaching a cell density of approximately 60%, the cells were divided into two groups (*n* = 3): one with 10 µM of Sor in the medium and one without. Each group was treated sequentially as follows: Group 1: Without gel immersion/irradiation; group 2: Immersion in IFBM gel for 3 min for cell treatment; group 3: 1.2 W/cm^2^ NIR irradiation of IFBM gel for 3 min for cell treatment. The cells were subsequently cultured for an additional period of time for detection. The treated cells were processed using EDU, ROS, and JC1 kits, respectively, according to the manufacturer’s instructions. They were subsequently observed and photographed using a fluorescence microscope, and the images were analyzed using ImageJ software.

### Biological transmission electron microscopy imaging

Cultured cells were initially seeded and grown in 24-well plates for 24 h, followed by subsequent treatments. The control group was incubated for an additional 24 h. The INS group underwent treatment by irradiating cells with 1.2 W/cm^2^ NIR of IFBM gel for 3 min, and Sor was added to the medium to a final concentration of 10 µM before continuing to incubate for 24 h. The cells were subsequently fixed for bio-transmission electron microscopy imaging analysis.

### GSH and MDA assay of cells

The cultured cells were seeded in 24-well plates and incubated for 24 h. Upon reaching an approximate cell density of 60%, the cells underwent the following treatment (*n* = 3): Group 1: No treatment; group 2: 1.2 W/cm^2^ NIR irradiation of IFBM gel for 3 min; group 3: Sor was added to the medium to a final concentration of 10 µM; group 4: 1.2 W/cm^2^ NIR irradiation of IFBM gel for 3 min, and Sor was added to the medium to a final concentration of 10 µM. The cells were subsequently cultured for an additional duration before processing and analysis using GSH or MDA kits.

### WB

Initially, the cultured cells were seeded into 24-well plates and incubated for 24 h. Upon reaching an approximate cell density of 60%, the cells were subjected to the following treatment (*n* = 3): Group 1: Control group without any treatment. Group 2: 1.2 W/cm^2^ NIR irradiation of ICG gel for 3 min; group 3: FBM gel immersion for 3 min; group 4: Sor was added to the medium to a final concentration of 10 µM; group 5: 1.2 W/cm^2^ NIR irradiation of IFBM gel for 3 min; group 6: 1.2 W/cm^2^ NIR irradiation of IFBM gel for 3 min, and Sor was added to the medium to a final concentration of 10 µM. Furthermore, groups 1–6 were subsequently cultured for 24 h before performing WB experiments utilizing standard procedures.

### Animal husbandry

Specific pathogen-free-grade Balb/c mice (4 weeks old, male) were obtained by the Anhui Provincial Laboratory Animal Center (Hefei, Anhui, China). All mice were maintained at 22–26 °C with suitable humidity and a 12-h light/dark cycle and were allowed to acclimatize for one week before subsequent experiments. All animal handling and protocols were approved by the Animal Ethics Committee of Anhui Medical University.

### *In vivo* photothermal effect and antitumor evaluation

*In vivo* photothermal effect: 0.1 mL of blank gel, FBM gel, ICG gel, and IFBM gel were prepared and subcutaneously injected into the mice (*n* = 3). The mice were subsequently irradiated with 1.2 W/cm^2^ NIR, and thermographic images were recorded at regular intervals.

*In vivo* antitumor effect: CT26 cells were cultured and subcutaneously injected at 5 million cells per mouse on day − 7 to induce tumorigenesis. On day 0, the mice were randomly divided into six groups for subsequent treatment (*n* = 5): (1) Control group: No treatment; (2) ICG gel + NIR group: Intratumoral injection of ICG gel on day 1, followed by photothermal treatment utilizing 1.2 W/cm^2^ NIR for 3 min on days 1, 6, and 11; (3) FBM gel group: Intratumoral injection of FBM gel on day 1, followed by no treatment; (4) Sor group: 0.1 mL of 10 µM Sor was intraperitoneally injected on days 0, 5, and 10; (5) IFBM gel + NIR group: Intratumoral injection of IFBM gel on day 1, followed by photothermal treatment with 1.2 W/cm^2^ NIR for 3 min on days 1, 6, and 11; (6) INS group: 0.1 mL of 10 µM Sor was injected intraperitoneally on days 0, 5, and 10, followed by intratumoral injection of IFBM gel on day 1 and photothermal treatment with 1.2 W/cm^2^ NIR for 3 min on days 1, 6, and 11. Tumor volume was measured and recorded at regular intervals throughout the experiment. The mice were euthanized on day 18, and tumor tissues, the heart, liver, spleen, lungs, and kidneys were harvested and processed following standard experimental procedures. Tumor tissues were photographed. Sections of major organs were analyzed using H&E staining, and tumor tissue sections were analyzed by H&E, TUNEL, and Ki67 immunohistochemistry. Images of the tissue sections were observed and captured using a Leica vertical microscope.

### Immune activation ability

Sequencing analysis: Tumor tissues from control and INS group mice were collected and submitted for eukaryotic transcriptome sequencing and analysis.

Flow cytometry: Tumor tissues and spleens from mice in different groups were collected and analyzed using flow cytometry for DC cells and T cells following standard experimental protocols (*n* = 3). For DC cells, APC-labeled CD80 surface antibody and PE/Cyanine7-labeled CD86 surface antibody were utilized. For T cells, FITC-labeled CD4 surface antibody, PE-labeled CD8 surface antibody, PE/Cyanine7-labeled CD44 surface antibody and APC-labeled CD62L surface antibody were utilized.

The obtained data were processed utilizing FlowJo software (Tree Star, Ashland).

mIHC: Tumor tissue sections from mice in various groups were collected and processed following standard experimental procedures. The sections were stained with DAPI and antibodies against CD8, CD11, and MHC II and photographed utilizing a Leica vertical microscope.

### Antitumor metastasis and recurrence ability

*In vivo* antitumor metastasis effect: Tumorization was performed utilizing 5 million CT26 cells per mouse. Subcutaneous tumorigenesis was performed on the right buttock dorsum of the mice on day − 9 and on the left buttock dorsum on day − 6. A liver metastasis model was created on day − 3. The mice were subsequently divided randomly into two groups for subsequent treatments (*n* = 4): (1) Control group: no treatment; (2) INS group: 0.1 mL of 10 µM Sor was injected intraperitoneally on days 0, 5, and 10. On day 1, IFBM gel was injected into the right tumor of the mice, followed by photothermal treatment with 1.2 W/cm^2^ NIR for 3 min on days 1, 6, and 11. Tumor volumes of the mice were measured and recorded periodically throughout the experiment. The mice were euthanized on day 18, and tumor tissues, livers, and spleens were harvested. After photographing the tumor tissues and livers, H&E staining was performed using standard experimental protocols, and tissue sections were observed and photographed using a Leica vertical microscope.

Flow cytometry: Tumor tissues and spleens from mice in different groups were collected for flow cytometric analysis of DC cells and T cells following standard experimental procedures (*n* = 3). For DC cells, APC-labeled CD80 surface antibody and PE/Cyanine7-labeled CD86 surface antibody were utilized. For T cells, FITC-labeled CD4 surface antibody, PE-labeled CD8 surface antibody, PE/Cyanine7-labeled CD44 surface antibody and APC-labeled CD62L surface antibody were utilized. The data were processed using FlowJo software (Tree Star, Ashland).

### Verification of the antitumor ability of ferroptosis

Ferrostain-1 (Fer-1) was selected as a specific inhibitor of ferroptosis for subsequent experiments. *In vitro* experiments: The cultured cells were seeded in 24-well plates and incubated for 24 h. Subsequent experiments were performed when the cell density reached approximately 60%.

CCK-8 experiments: Cells were treated as follows and were incubated for 24 h before performing absorbance assays and analyzed utilizing CCK-8 according to the instructions (*n* = 4): Group 1: No treatment; group 2: Cells were irradiated with IFBM gel for 3 min using 1.2 W/cm^2^ NIR, and Sor was added to the culture medium to achieve a final concentration of 10 µM; group 3: 2 mM of Fer-1 solution was added to the culture medium to achieve a final concentration of 2 µM; group 4: Cells were treated as in group 2, with 2 mM of Fer-1 solution added to the culture medium to achieve a final concentration of 2 µM.

GSH and MDA assay: Cells were treated as follows and incubated for the indicated periods. Subsequently the cells were processed and analyzed using commercial GSH or MDA kits (*n* = 3): Group 1: No treatment; group 2: Cells were irradiated with IFBM gel and 1.2 W/cm^2^ NIR for 3 min, and Sor was added to the culture medium to achieve a final concentration of 10 µM; group 3: 2 mM of Fer-1 solution was added to the culture medium to achieve a final concentration of 2 µM as the same treatment as group 2.

*In vivo* experiments: CT26 cells were cultured, and on day − 9, subcutaneous tumor formation was performed by subcutaneously injecting 5 million CT26 cells per mouse. The mice were randomly divided into three groups for subsequent treatments on day 0.

*In vivo* antitumor effects (*n* = 4): Group 1: Without treatment; group 2: Intraperitoneal injection of 0.1 mL of 10 µM Sor on days 0, 5, and 10, and intratumoral injection of IFBM gel on day 1, followed by photothermal treatment utilizing 1.2 W/cm^2^ NIR for 3 min on days 1, 6, and 11; group 3: received the same treatment as (group 2), 50 µg/mL Fer-1 solution was injected intraperitoneally at a dose of 10 mg/kg 2 h before each intraperitoneal injection of Sor.

The tumor volumes of the mice were measured and recorded periodically throughout the experiment. The mice were euthanized on day 18 for the collection of tumor tissues and spleens. Tumor tissues were photographed, fixed, and processed for H&E staining following standard experimental procedures. The tissue sections were observed and photographed using a Leica vertical microscope.

Flow cytometry: Tumor tissues and spleens from different groups of mice were collected for flow cytometry analysis of DC cells and T cells following standard experimental procedures (*n* = 3). For DC cells, APC-labeled CD80 surface antibody and PE/Cyanine7-labeled CD86 surface antibody were utilized. For T cells, FITC-labeled CD4 surface antibody, PE-labeled CD8 surface antibody, PE/Cyanine7-labeled CD44 surface antibody and APC-labeled CD62L surface antibody were utilized. The data were processed using FlowJo software (Tree Star, Ashland).

### Statistical analysis

All statistical analyses for gel characterization were based on independently prepared batches, with repeated measurements of each batch treated as technical replicates and averaged prior to analysis, unless otherwise specified. Statistical analyses for *in vitro* experiments were primarily analyzed based on technical replicates, with values averaged prior to statistical analysis, unless otherwise specified. Statistical analyses for *in vivo* experiments were based on biological replicates, with technical replicates averaged prior to analysis, unless otherwise specified. Experimental data are presented as the mean ± standard deviation (SD). The statistical significance was analyzed using Student’s t-test using OriginPro 2021 software. A *p* < 0.05 was considered statistically significant (*p < 0.05, **p < 0.01, ***p < 0.001, and ****p < 0.0001).

## Supplementary Information

Below is the link to the electronic supplementary material.

Supplementary Material 1

## Data Availability

No datasets were generated or analysed during the current study.
