# Supplementary material for: Ferroptosis-driven in situ vaccine-like antitumor effects: NIR-triggered IFBM hydrogel synergizes with sorafenib to unleash systemic antitumor immunity
Source: J Nanobiotechnology. 2026 Mar 8;24:362. doi: 10.1186/s12951-026-04262-z (PMC13081275; doi:10.1186/s12951-026-04262-z)
Supplement: Supplementary file 1 — Supplementary Material 1 [file 12951_2026_4262_MOESM1_ESM.docx]

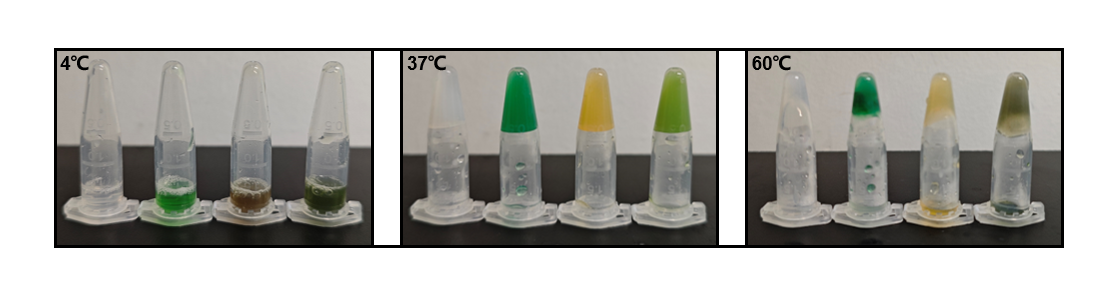


Fig. S1. Photographs of different component hydrogels at 4℃, 37℃, and 60℃, from left to right, Gel, ICG Gel, FBM Gel, and IFBM Gel.


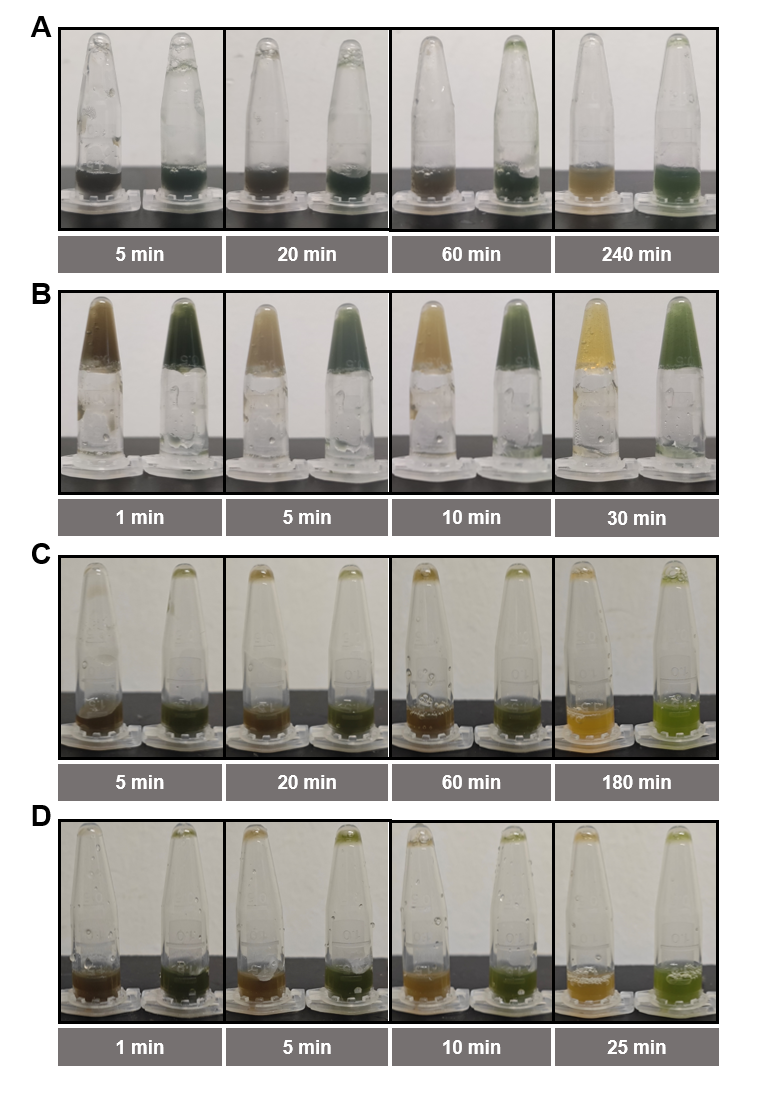


Fig. S2. A) & B) The photographs of the degradation of different components of hydrogels at 4℃ & 37℃, FBM Gel on the left and IFBM Gel on the right. C) and D) The photographs of the degradation of suspensions of different compositions at 4℃ and 37℃, FBM NCs suspension on the left and IFBM NCs suspension on the right.


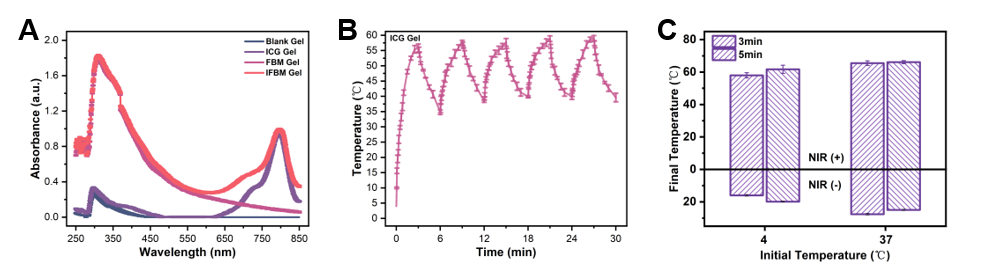


Fig. S3. A) Spectral absorption curves of different component hydrogels. B) Temperature-time change curves of 50 μg/mL ICG gel under 1.2 W/cm^2^ NIR on-off irradiation. C) Effect of initial temperature and room temperature on the photothermal temperature of ICG Gel (n = 3, mean ± SD).


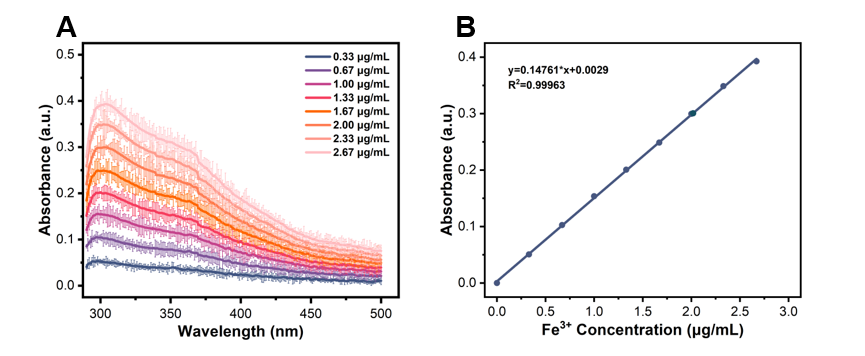


Fig. S4. A) Spectral analysis of Fe^3+^ solutions with different concentrations. B) Absorption peak-concentration fitting curves of Fe^3+^ solution (n = 3, mean ± SD).


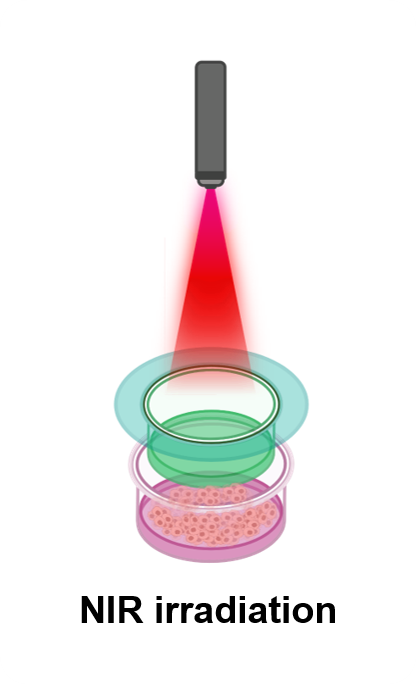


Fig. S5. Schematic diagram of cellular photothermal treatment.


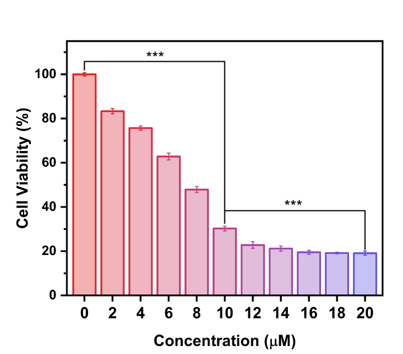


Fig. S6. Effect of Sor concentration on the activity of CT26 cells (n = 4, mean ± SD).


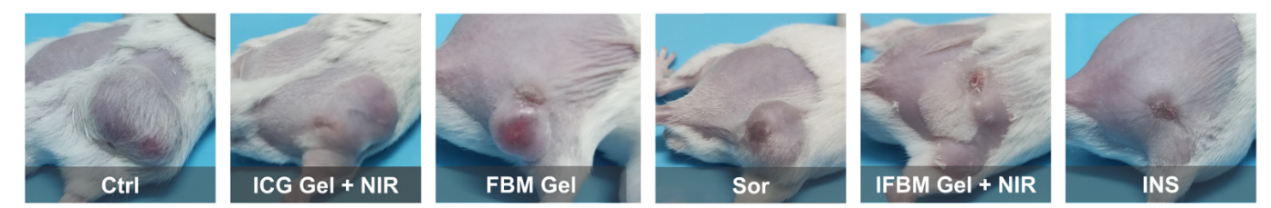


Fig. S7. Photographs of different groups of mice at day 18.


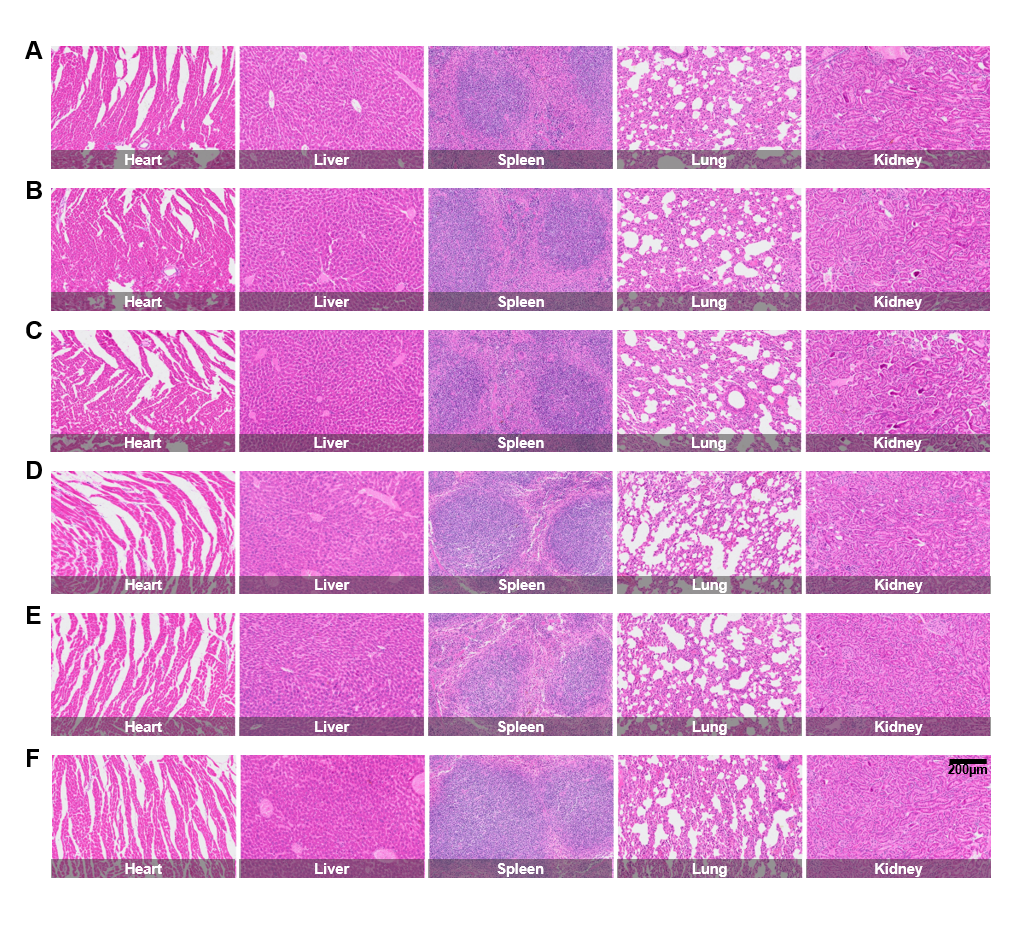


Fig. S8. A-F) H&E staining analysis of major organ sections from different groups of mice. (A: Ctrl Group, B: ICG Gel + NIR Group, C: FBM Gel Group, D: Sor Group, E: IFBM Gel + NIR Group, F: INS group).


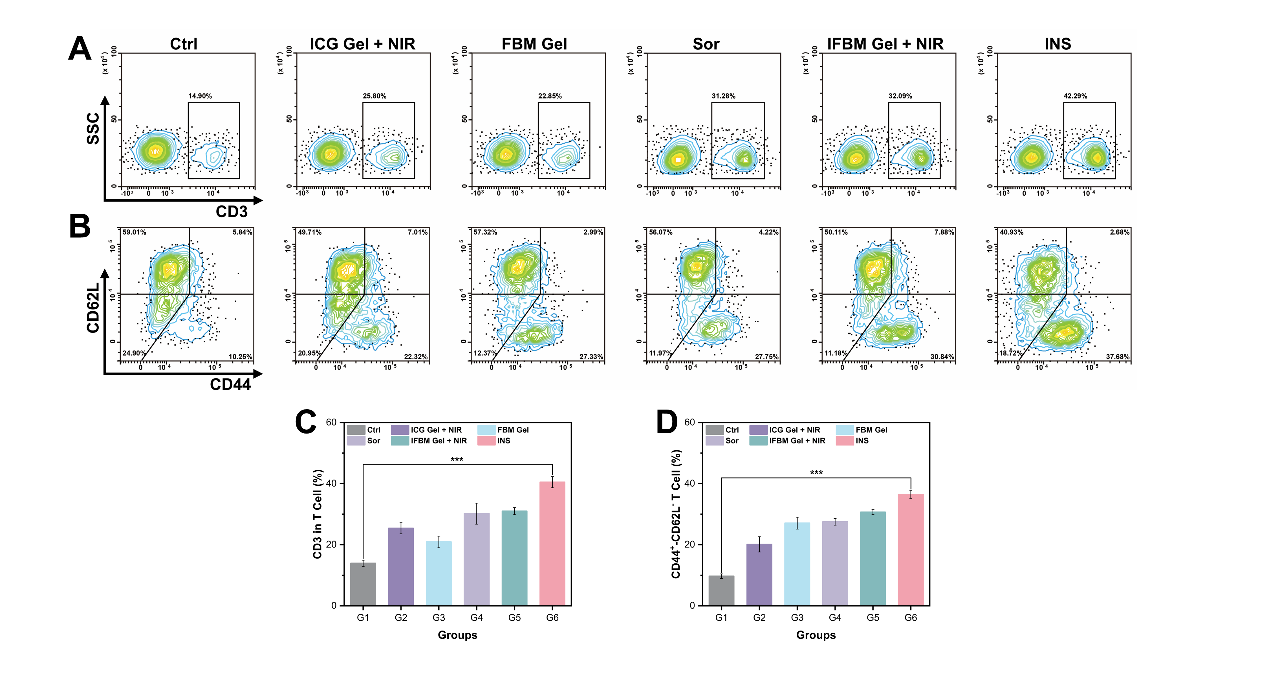


Fig. S9. Spleen T cell flow cytometry plots of different groups of CD3 A) & CD44, CD62L B), C) and D) corresponding quantitative plots (G1: Ctrl group, G2: ICG gel + NIR group, G3: FBM gel group, G4: Sor group, G5: IFBM gel + NIR group, G6: INS group).


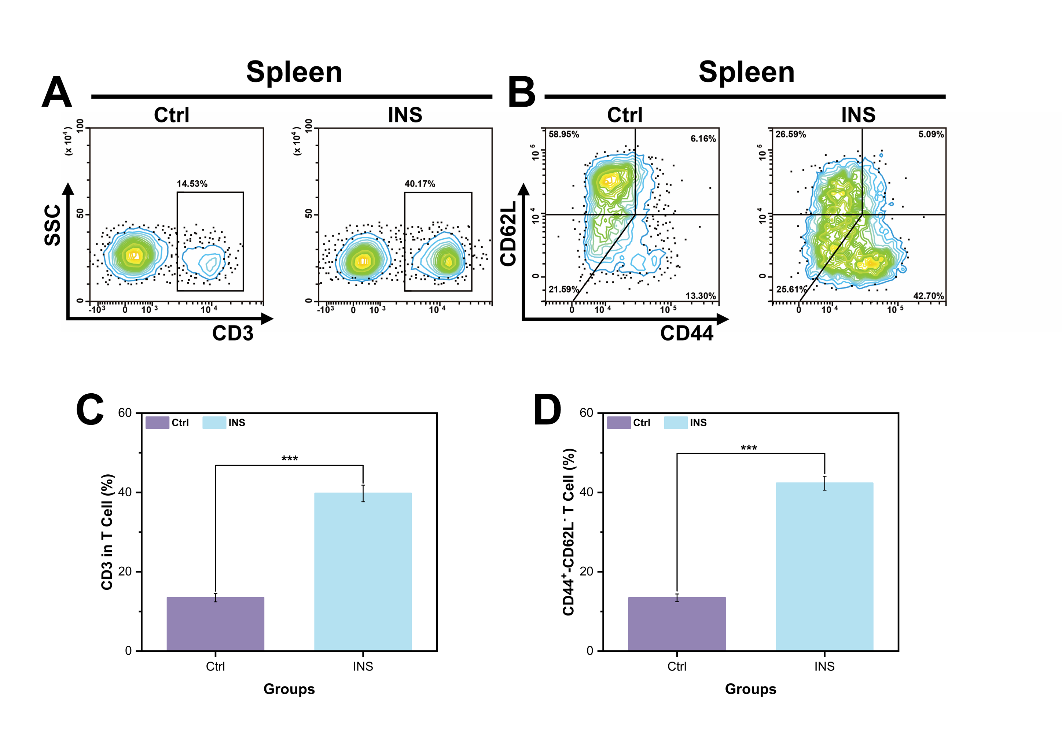


Fig. S10. Spleen T cell flow cytometry plots of different groups of CD3 A) & CD44, CD62L B), C) and D) corresponding quantitative plots (G1: Ctrl group, G2: INS group).


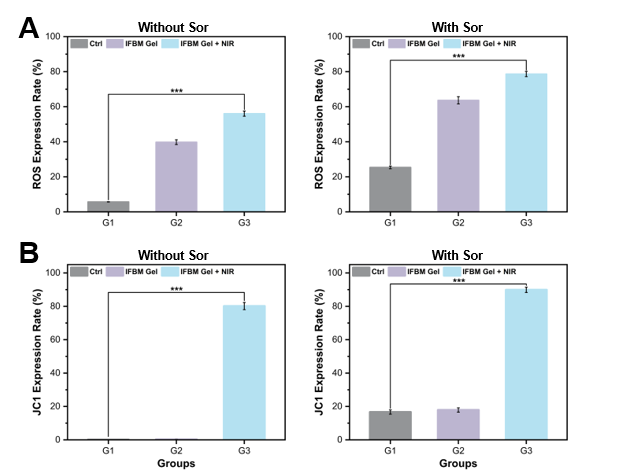


Fig. S11. A) Quantitative analysis of ROS in CT26 cells under different group treatments. B) Quantitative analysis of JC1 in CT26 cells under different group treatments (n = 3, mean ± SD).


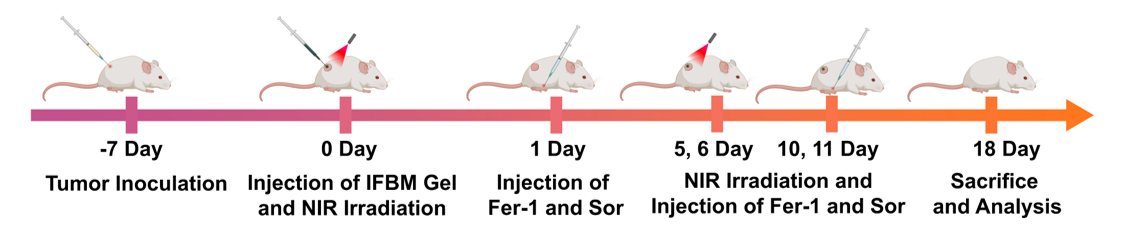


Fig. S12. Flow chart of the ferroptosis inhibition assay in vivo.


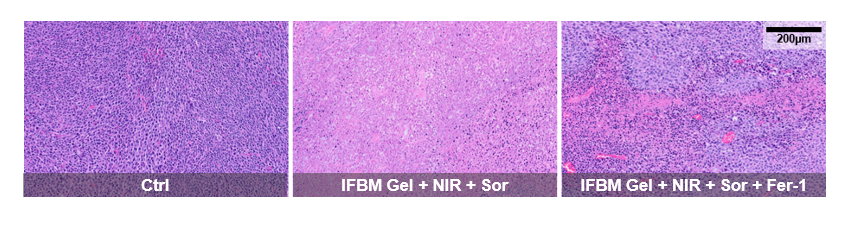


Fig. S13. H&E staining analysis of tumor sections of different groups of mice.


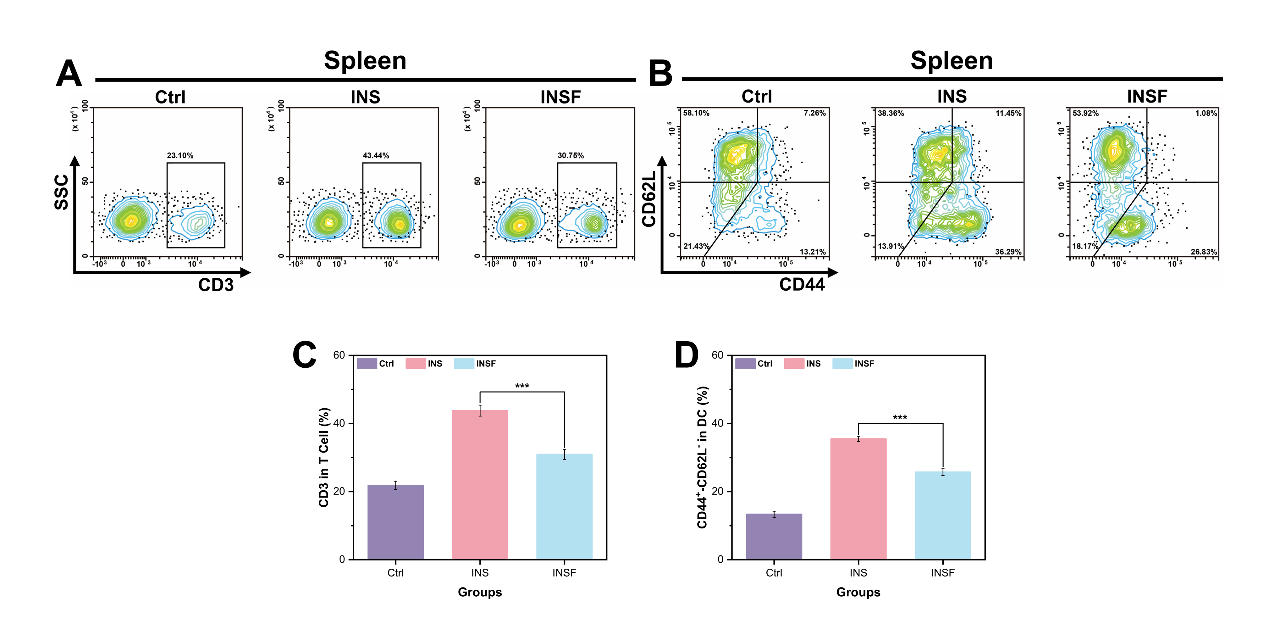


Fig. S14. Spleen T cell flow cytometry plots of different groups of CD3 A) & CD44, CD62L B), C) and D) corresponding quantitative plots (G1: Ctrl group, G2: INS group, G3: INSF group).
